# LOCO: The 88-million-word language of conspiracy corpus

**DOI:** 10.3758/s13428-021-01698-z

**Published:** 2021-10-25

**Authors:** Alessandro Miani, Thomas Hills, Adrian Bangerter

**Affiliations:** 1 Institute of Work and Organizational Psychology, University of Neuchâtel, Rue Emile-Argand 11, 2000 Neuchâtel, Switzerland; 2 Department of Psychology, University of Warwick, University Road, Coventry, CV47AL UK; 3 The Alan Turing Institute, British Library, 96 Euston Road, London, NW1 2DB UK

**Keywords:** ■■■

## Abstract

The spread of online conspiracy theories represents a serious threat to society. To understand the content of conspiracies, here we present the language of conspiracy (LOCO) corpus. LOCO is an 88-million-token corpus composed of topic-matched conspiracy (*N* = 23,937) and mainstream (*N* = 72,806) documents harvested from 150 websites. Mimicking internet user behavior, documents were identified using Google by crossing a set of seed phrases with a set of websites. LOCO is hierarchically structured, meaning that each document is cross-nested within websites (*N* = 150) and topics (*N* = 600, on three different resolutions). A rich set of linguistic features (*N* = 287) and metadata includes upload date, measures of social media engagement, measures of website popularity, size, and traffic, as well as political bias and factual reporting annotations. We explored LOCO’s features from different perspectives showing that documents track important societal events through time (e.g., Princess Diana’s death, Sandy Hook school shooting, coronavirus outbreaks), while patterns of lexical features (e.g., deception, power, dominance) overlap with those extracted from online social media communities dedicated to conspiracy theories. By computing within-subcorpus cosine similarity, we derived a subset of the most representative conspiracy documents (*N* = 4,227), which, compared to other conspiracy documents, display prototypical and exaggerated conspiratorial language and are more frequently shared on Facebook. We also show that conspiracy website users navigate to websites via more direct means than mainstream users, suggesting confirmation bias. LOCO and related datasets are freely available at https://osf.io/snpcg/.

**Supplementary Information:**

The online version contains supplementary material available at 10.3758/s13428-021-01698-z.

## Introduction

Conspiracy theories (CTs) are narratives that attempt to explain significant social events as being secretly plotted by powerful and malicious elites at the expense of an unwitting population (Douglas et al., 2019; Samory & Mitra, 2018b). Belief in CTs is widespread. In 2013, it was estimated that over 50% of the US population believed in at least one CT (Oliver & Wood, 2014), while in 2020, in the middle of the COVID-19 pandemic, health-related misinformation attracted four times as much traffic as official health sources on social media (AVAAZ, 2020). The consequences associated with the circulation of such theories are not trivial, potentially leading to detrimental social action (Franks et al., 2013; Imhoff et al., 2021; Sternisko et al., 2020). Belief in CTs is linked to rejection of official information and science (Raab, Auer, et al., 2013a; Raab, Ortlieb, et al., 2013b; van der Linden, 2015), decreased intentions to adopt vaccines (Jolley & Douglas, 2014b; Lazarus et al., 2020; Salmon et al., 2005), resistance to COVID-19 containment measures and vaccination (Biddlestone et al., 2020; Lazarus et al., 2020), and reduced protection against sexually transmitted diseases (Bogart et al., 2010). CT belief is also related to general distrust and political alienation along with endorsement of nonnormative (vs. normative) political intentions (Einstein & Glick, 2015; Imhoff et al., 2021; Jolley & Douglas, 2014a). Such beliefs also provide justification for engaging in everyday crime (Jolley et al., 2019; Jolley & Paterson, 2020) and anti-Semitic and Islamophobic attitudes (Golec de Zavala & Cichocka, 2012; Swami et al., 2018). Therefore, within psychology, research has typically focused on motivational and contextual factors as well as individual differences underlying belief in CTs (Butter & Knight, 2020; Douglas et al., 2019; Douglas & Sutton, 2018).

A more complete understanding of CTs requires understanding how they spread. The current focus on individual beliefs, predispositions, and biases is of limited utility in this respect, for two reasons. First, beliefs are not straightforwardly connected to CT transmission. For example, a skeptical-minded individual may share a CT document within a debunking community for critical purposes (Franks et al., 2017), or a credulous individual may hesitate to share such a document in a science-oriented community for fear of being stigmatized (Lantian et al., 2018). Moreover, transmission can also be motivated strategically, independently of belief, to influence constituencies, such as when CTs or fake news is intentionally shared on social media to affect outcomes like voter behavior (Bangerter et al., 2020; Douglas et al., 2019). Second, CT beliefs do not spread per se. Rather, CTs spread as materialized forms of belief, conveyed as narratives in the form of written text (e.g., from webpages or social media posts), video (e.g., from video-sharing platforms such as YouTube), images (e.g., internet memes), or eventually audio (e.g., podcasts or the recent audio-based social media Clubhouse). Regardless of their form, CT beliefs emerge in the minds of recipients when they interact with such content. For whatever reasons CT narratives are created, they circulate, sticking in the mind of conspiracy-predisposed recipients and potentially motivating individual and collective action (Franks et al., 2013; Imhoff et al., 2021; Jolley & Paterson, 2020). Therefore, to understand the spread of CTs and their outcomes, research should investigate the content of CT narratives.

On the internet, misinformation spreads faster, farther, and deeper within groups of like-minded individuals (Del Vicario et al., 2016a, b; Vosoughi et al., 2018, but see Clarke, 2007; Uscinski et al., 2018 for a critical view). The internet constitutes a system of information proliferation by which many people form opinions in regard to political parties, social issues, and health-related information (Betsch et al., 2011). In the Web 2.0 version of the internet, information is produced and consumed in a horizontal fashion, allowing anyone to create and share content, with few editorial filters (Aupers, 2012; Bessi et al., 2015a, b). CT texts may thus have the same epistemological weight for many users as mainstream texts, and compete with them for attention (Bessi et al., 2014; Eicher & Bangerter, 2015; Hills, 2019). The perceived credibility of epistemic sources is also a function of belief in CTs (Imhoff et al., 2018). This makes epistemic authority difficult to evaluate, especially when conspiratorial narratives are promoted by political leaders (Barkun, 2017), by scholars in prestigious journals (Wakefield et al., 1998), and by Nobel Prize winners (Perez & Montagnier, 2020).

Research on the content and circulation of CTs on the internet has focused on user-generated texts such as comments and posts gathered from social media such as Twitter (Mitra et al., 2016; Wood, 2018), Facebook (Bessi, 2016; Bessi, Zollo, et al., 2015b; Brugnoli et al., 2019; Smith & Graham, 2019), Reddit (Klein et al., 2018, 2019; Samory & Mitra, 2018a, 2018b), Gab (Zannettou et al., 2018), or comment sections of news websites (Wood & Douglas, 2013, 2015). This approach has the advantage of exploring large, ecologically valid samples of text as a complement to psychological investigations of CT beliefs. However, it is difficult to reliably extract measures of individual belief from comments embedded within the noisy and heterogeneous discussion threads of conspiracy believers and debunkers (Wood & Douglas, 2013, 2015; but see Klein et al., 2019). Moreover, discussion threads limit the utility of extracted text because the comments and posts are brief, are contextualized in the discussion in which they are embedded, and are incapable of spreading independently from the whole thread. As a matter of fact, discussion threads are not conspiracy narratives per se. While the comments and posts they contain might instill curiosity, reinforce existing beliefs, or support conversion, they often do not constitute the actual source through which CTs are transmitted.

### Towards corpora of CT texts

One valuable source of CTs for academic research aimed at understanding the content and transmission of CTs is CT websites. Although social media sites engage more traffic and are overall more popular than other websites (Facebook, Twitter, and Instagram are respectively ranked as the third, fourth, and fifth most popular websites following Google and YouTube, according to similarweb.com, accessed on 20 March 2021), websites provide more in-depth and elaborated discourse than posts and tweets on social media, which are nevertheless crucial for the spread of webpages. Conspiracy websites, specifically, are specialized sources created for the purpose of developing, collecting, and spreading CTs. These websites provide ample page space to showcase arguments that discredit official narratives and function as trustworthy epistemic sources for CT believers. Analysis of CT webpages offers a series of advantages. Webpages constitute standalone, structured texts nested within sources (i.e., websites) and as such are accompanied by paratextual (i.e., metadata) information. As standalone documents, webpages can easily be shared on social media and so can provide measures of spread. Appropriately identified webpages, therefore, would be beneficial for studying the content of CTs, and a large corpus of such CTs could provide a solid grounding for CT research.

Only a handful of studies, focused on related phenomena such as anti-vaccine movements, rumors, and fake news, have built corpora from online material (discussed below). Yet, to our knowledge, the field lacks a corpus specifically focused on online CTs. In this section, we describe published work, stressing its strengths and weaknesses.

General-purpose linguistic corpora such as the WaCky corpus (Baroni et al., 2009) and the British National Corpus (BNC, Aston & Burnard, 1998), although composed of large collections of texts, do not generally allow researchers to focus on either the source type (e.g., conspiracy vs. mainstream) or a specific topic (e.g., an event that has generated a CT). It should be noted, however, that documents in WaCky were gathered from a set of 2000 seeds consisting of randomly chosen pairs of content words selected from the BNC, meaning that seeds were used as keywords to retrieve webpages. This represents a useful approach which we use here: by creating ad hoc seeds, data collection can be directed to a particular (set of) topic(s) encapsulated in the seed.

Other corpora focus on specific themes. In the field of online anti-vaccine movements, a few studies have collected webpages gathered through search engines (Fu et al., 2016; Okuhara et al., 2017; Sak et al., 2015). This approach is convenient, as it allows researchers to obtain data by mimicking how users retrieve information. However, because these corpora were collected manually, sample sizes are limited, therefore reducing generalizability. To a different degree, the CORPS corpus (Guerini et al., 2013) is composed of 3600 political speeches gathered from the web. Nonetheless, being composed of only one genre without any (matched) control group, CORPS does not enable comparisons beyond descriptive analyses.

Focusing on rumors and fake news, other studies have built corpora that include a control group that allows between-group comparisons (Castelo et al., 2019; Kwon et al., 2017). Kwon et al. (2017), for example, built two Twitter subcorpora from a list of rumor and non-rumor events (henceforth RumTweet). Castelo et al. (2019), on the other hand, collected material (fake news vs. mainstream) via lists of reliable and unreliable websites compiled by independent fact checkers (henceforth FNweb). This approach is useful because it reduces selection bias during data collection. However, although these two studies allow us to narrow the sampling method to obtain either two-group sources (websites) or two-group themes (rumors), they present an important limitation. In fact, to systematically study phenomena related to language (e.g., conspiracy, or fake news, or rumors) through a corpus, many forms of analysis are likely to require subcorpora matched by topic. This allows researchers to compare different versions of the same event to identify discriminating features. If not matched, the two subcorpora are treated as bags of words (as in Castelo et al., 2019, and Kwon et al., 2017), ignoring the inherent structure emerging from different themes or sources. Although some changes are expected to emerge systematically from a bag-of-words approach, there may also be important topic-specific differences. For example, differences between CT and mainstream accounts of Princess Diana’s death are likely to differ in informative ways from CT and mainstream accounts of COVID-19. These differences can only emerge from a topic-matched corpus.

Overcoming this limitation, the PHEME dataset (Zubiaga et al., 2016) focuses on predefined events that have generated rumors, allowing researchers to compare rumor with non-rumor tweets around specific events. Yet, the only work, to our knowledge, that has focused on CTs is that of Uscinski and collaborators (2011), who gathered 100,000 published letters to the editor of *The New York Times* (henceforth NYT) from 1897 to 2010. After the data were collected, each document was manually coded as either referring to a conspiracy or not, of which 800 were identified as conspiracies. In addition, differently from the other works we reviewed above, the authors coded several groups of actors post hoc (e.g., left/right/foreign political actors, capitalists/communists, media, government institutions, and other).

Here, we present LOCO**,**^1^ our Language Of COnspiracy corpus that was built upon the strengths and weaknesses of the reviewed corpora.

Table 1 shows the corpora comparison, including LOCO, and summarizes each corpus’ key features, including the focus (i.e., the goal, e.g., general-purpose language or fake news), the source of the material gathered (e.g., from webpages or twitter), size expressed in number of documents and tokens (i.e., non-unique words), presence of topics (e.g., events or themes) or grouping (e.g., rumors vs. non-rumors) structures, the date range of documents expressed in years, and whether the material is freely available.

**Table 1:** Key features of eight corpora relevant to conspiracy theory content

Resource	BNC	WaCky	CORPS	FNweb	RumTweet	PHEME	NYT	LOCO
Focus	Language	language	Political speeches	Fake news	Rumors	Rumors	Conspiracy	Conspiracy
Obtained from	Printed material	Web pages	Web pages	Webpages from list of websites	Twitter	Twitter	Newspaper	Webpages from list of websites
Number of documents	4 K	2.69 M	3.6 K	14 K (7 K fake)	192 K tweets (61 K rumor)	7.5 K threads (35 K rumor tweets)	100 K (800 conspiracy)	96 K (24 K conspiracy)
Number of tokens	100 M	1.9 B	7.9 M	7 M*	2.8 M*	100 K*		88 M
Topic structure	NO	2 K seeds	NO	NO	YES 111 events (60 rumors, 51 non-rumors)	YES 9 events	YES	YES 47 seeds 600 topics
Grouping structure	NO	NO	NO	YES	YES	YES (matched)	YES	YES (matched)
Year range			1917 2010	2013 2018	2006 2009	Events around 2014–2015	1897 2010	1853 2020
Freely available	YES	YES	YES	YES	YES	YES	NO	YES

*Note*. Resources: BNC (Aston & Burnard, 1998); WaCky (Baroni et al., 2009); CORPS (Guerini et al., 2013); FNweb (Castelo et al., 2019); RumTweet (Kwon et al., 2017); PHEME (Zubiaga et al., 2016); NYT (Uscinski et al., 2011). *Number of tokens calculated from studies’ freely available datasets

## The language of conspiracy (LOCO) corpus

LOCO is a multilevel topic-matched corpus composed of standalone documents (*N* = 96,743) gathered via ready-made lists of conspiracy and mainstream websites (see LOCO’s key feature in Table 2). LOCO has been built as a freely available text source from which researchers can extract features and/or generate predictive and classification models. Previous studies of CT textual data have extracted lexical features (Del Vicario, Vivaldo, et al., 2016b; Faasse et al., 2016; Klein et al., 2019; Mitra et al., 2016; Samory & Mitra, 2018a; Wood & Douglas, 2015), topic distributions (Bessi, Zollo, et al., 2015b; Klein et al., 2018; Mitra et al., 2016; Samory & Mitra, 2018b), and narrative patterns (Samory & Mitra, 2018b). Such analyses can be replicated and extended with LOCO due to its rich metadata.

**Table 2:** Summary statistics of mainstream, conspiracy, and all documents in LOCO

	Mainstream	Conspiracy	Whole corpus
No. of documents	72,806	23,937	96,743
No. of websites	92	58	150
Range of years	1853–2020	2004–2020	1853–2020
	*M* (*SD*) [range]	*M* (*SD*) [range]	*M* (*SD*) [range]
No. of words per document	805.94 (939) [97–9507]	1236.32 (1307) [100–9428]	912.43 (1059) [97–9507]
*Total no. of words*	*58,677,322*	*29,593,678*	*88,271,000*
No. of sentences per document	37.92 (47.89) [1–1087]	59.63 (69.58) [1–1047]	43.29 (54.88) [1–1087]
*Total no. of sentences*	*2,760,789*	*1,427,397*	*4,188,186*
No. of paragraphs per document	16.56 (19.30) [1–829]	24.51 (32.83) [1–905]	18.53 (23.64) [1–905]
*Total no. of paragraphs*	*1,205,904*	*586,748*	*1,792,652*

The main goal of LOCO is to shed light on the language of conspiracy. To this aim, LOCO is built on documents that revolve around CTs. Because we do not yet know what the language of conspiracy is, i.e., to what extent conspiracy language differs from non-conspiracy language, selecting documents (e.g., from webpages) based on an a priori definition of conspiracy would be difficult. At best, selecting documents based on their content would result in both a limited sample size (due to manual coding, see e.g. Fu et al., 2016; Okuhara et al., 2017; Sak et al., 2015) and limited heterogeneity (due to selection criteria based on a specific linguistic/rhetoric style). We therefore chose to categorize document selection starting from the source (i.e., websites).

Not all content from conspiracy websites will contain CTs. Intuitively, it is unlikely that all ~93,000 webpages in www.globalresearch.ca contain CTs, and some content might come from neighboring genres such as rumors, fake news, urban legends, and pseudoscience. To provide an estimate of how well conspiracy and mainstream documents reflect their true labels and how well the two sources can be distinguished from each other, we have blindly coded a subset of LOCO’s documents (60 documents from each subcorpus, see Section SM1 in supplemental material) as being either conspiratorial or not. With an overall accuracy of .88 (Cohen’s *k* = .77), we have correctly classified as conspiracy 85% of documents and correctly classified as mainstream 92% of documents. The lower classification performance on conspiracy documents suggests that not all documents from conspiracy websites are in fact CTs, while mainstream documents are less ambiguously classified as non-conspiracy. An alternative explanation is that conspiracy texts are difficult to distinguish from mainstream texts, at least via human inspection (meaning that future algorithms might find features that help improve on human classification). These results also suggest that conspiracy and mainstream texts overlap to some extent (suggesting a continuum).

The multilevel structure of LOCO allows us to take into consideration natural hierarchical grouping of documents cross-nested within websites and topics. At the document, webpage, and website levels, LOCO’s metadata^2^ allow researchers to create subsets of documents or to add covariates during analyses. In Table 3, we summarized the key variable types we provide with LOCO for each level. For example, each document is associated with topic labels that summarize its semantic content. These labels refer to the topics that have the highest probability (among all topics extracted from LOCO) of describing the document’s content (see “Topic extraction” section). This is useful for tracking differences (e.g., in lexical features) between conspiracy and mainstream texts within a specific topic (e.g., Princess Diana’s death), within a set of related topics (e.g., coronavirus outbreak in China, coronavirus outbreak in the United States), between topics (e.g., Pizzagate vs. moon landing), or within and between topics, e.g., by using a 2 (e.g., Princess Diana, coronavirus) × 2 (conspiracy, mainstream) factorial design. Similar analyses can be performed using the data LOCO provides on website information about political bias, factual reporting, and website category. For most (~67%) webpages, we gathered information about their upload/creation date (see “Date” section). This allows researchers to test time-related hypotheses such as the evolution through time of topics or lexical features (e.g., coronavirus topics over time). Other crucial features of LOCO are the spread and popularity metrics associated with both websites and webpages. These metrics allow researchers to test hypotheses about social media transmission, for example, testing webpages’ spread and engagement while correcting for the website’s popularity. Last but not least, LOCO is provided with a set of almost 300 lexical features (e.g., psychological processes associated with words) derived from two widely used and validated text-analysis programs based on word-within-category counting.

**Table 3:** Types of variables included in LOCO

Level	Variable type	Example of variable	Section
1. Document	Raw content	Document ID	Table 6
		Title	3.4
		Text	3.4
	Features	Number of words, sentences, paragraphs	3.8.2
	Semantic content	Topic	3.6
		Lexical features	3.5
	Conspiracy content	Representativeness	3.7
		Mention of conspiracy	3.8.1
2. Webpage	Information	Website host	3.2
		URL	3.3
		Date	3.8.3
		Seeds	3.1
	Spread	Facebook shares, comments, and reactions	3.8.4
3. Website	Classification	Political orientation, factual reporting, category	3.8.5
	Size	Number of webpages	3.8.6
	Popularity	Visits, traffic, and rank	3.8.6
	Spread	Facebook shares, comments, and reactions	3.8.4

## Method

### Seed selection

Similar to the construction of the WaCky corpus (Baroni et al., 2009), we used seeds (i.e., keywords) to retrieve the webpages that provide the texts for LOCO. Seeds were extracted from the items of two CT-based surveys: a national poll (Jensen, 2013, Source 1, e.g., “*Do you believe that Lee Harvey Oswald acted alone in killing President Kennedy, or was there some larger conspiracy at work?*”) and the 17-item “endorsement of conspiracy theories” from Douglas and Sutton (2011, Source 2, e.g., “*The American moon landings were faked*”). We extracted the seeds from these surveys for two reasons. Firstly, these surveys on CTs encompass a broad set of well-known CTs, since they are supposed to measure specific beliefs from a wide range of people. Secondly, these surveys condense each theory within a short space, usually a sentence. These two surveys were chosen because, while they measure specific theories, they are broad in scope, and encompass a large and heterogeneous set of CTs. Items from both surveys were grouped to obtain a unique seed (e.g., “*Princess Diana faked her own death so she and Dodi could retreat into isolation*,” “*Princess Diana’s death was an accident*,” and “*One or more rogue ‘cells’ in the British Secret Service constructed and carried out a plot to kill Princess Diana*” were merged as “Princess Diana’s death”).

We further broadened the pool of seeds by manually adding 20 seeds corresponding to popular (e.g., Illuminati, genetically modified organisms, Pizzagate) and current (e.g., coronavirus, Bill Gates, 5G) CTs missing from Sources 1 and 2. Note that seeds such as “chemtrails,” when applied to mainstream documents, in most if not all cases return documents referring to CTs. We keep these documents in LOCO so as to have a broad mainstream pool and allow users to create subsets of texts prior to analyses (e.g., by removing mainstream documents that mention CTs, see “Mentioning “Conspiracy” and Effect of mentioning conspiracy” sections). In order to include events that might be associated with different spellings, for some seeds we used synonyms (e.g., big pharma, drug companies, and pharmaceutical industry; new world order and NWO; climate change and global warming). In Table 4, we show the full set of seeds used to retrieve documents and the final document count in LOCO by source type. Note that the seed count is larger than the number of documents. This is because a single webpage can be returned by a Google search using different keywords. For example, if a document relates to Princess Diana’s death due to an Illuminati plot, then this document would be returned twice for both “Princess diana death” and “illuminati” searches.

**Table 4:** List of seeds

seed source	No. of conspiracy documents	No. of mainstream documents	
5g	m	702	1664
aids	2	1025	2428
alien	1, 2	813	1715
barack obama	1	496	1485
big foot	1	708	2019
big pharma	1	716	1758
bill gates	m	717	1623
cancer	m	839	2098
chemtrails	1	744	549
cia cocaine	1	552	1030
climate change	1, 2	889	2166
coronavirus	m	1104	2588
covid 19	m	1004	2395
drug companies	1	1024	2356
ebola	m	626	2140
elvis death	m	188	1386
elvis presley	m	132	1258
flat earth	m	605	1646
fluoride water	1	395	1384
george bush	1	844	1737
george soros	m	735	1178
global warming	1, 2	896	1793
gmo	m	620	1924
illuminati	m	804	1479
jfk assassination	1, 2	607	1344
jonestown suicide	2	42	594
mh370	m	167	1086
michael jackson death	m	616	1564
mind control	1	949	2036
moon landing	1, 2	349	1579
new world order	1	1036	2162
nwo	1	814	1350
osama bin laden	1	645	1415
paul mccartney death	1	149	1190
pharmaceutical industry	1	828	1684
pizzagate	m	359	1012
planned parenthood	m	626	1434
population control	m	972	2295
princess diana death	2	309	1338
reptilian	1	494	1418
saddam hussein	1	677	1623
sandy hook	m	470	1500
september 11 attack	1, 2	939	2207
vaccine	1	803	2125
vaccine autism	1	531	1654
vaccine covid	m	923	2031
zika virus	m	473	1675

*Note*. Sources 1, 2, and m refer to: 1 = Jensen (2013); 2 = Douglas and Sutton (2011), and m = manual

Note that although we used seeds as keywords to retrieve webpages, we do not intend seeds to serve as proxies for document content. This is because a webpage is returned by Google if the seed is present in the webpage (but note, not necessarily in the main text) at least once. The seed presence in the webpage, however, does not necessarily indicate that the seed reflects the main topic of the document’s text, because the seed can be contained in boilerplate texts or in the comments section of the webpage. Instead, we remind the user that for a more precise content of documents, we offer a more fine-grained measure of document content (extracted from the cleaned text), namely topics (see “Topic extraction” section). We include the seed variable in the LOCO dataset, believing it might be useful for answering other questions, e.g., regarding webpage indexing.

### Website lists

Following previous work (Pennycook & Rand, 2019), we gathered a list of conspiracy websites from mediabiasfactcheck (MBFC).^3^ Websites are labeled by MBFC as conspiracy if they publish unverifiable information related to known conspiracies such as the New World Order, Illuminati, false flags, aliens, anti-vaccination propaganda, etc. (for further details, see category descriptions in “Website category” section). From the whole list of 241 conspiracy websites, we selected (in December 2019) those that scored the highest on the conspiracy rating (i.e., “tin foil hat,” *N* = 68^4^). This increased the chances of obtaining highly conspiratorial texts, limiting contamination by mainstream or less conspiratorial texts.

The mainstream list of websites was created (in June 2020) in a data-driven fashion by extracting the websites returned by Google for each seed. While maximizing data acquisition, this approach also mimics users’ online behavior. We proceeded as follows. For each seed, we created a Google query, gathered the resulting top 40 URLs, and extracted the websites’ domains.^5^ We repeated this operation with different IPs, mimicking the searches from the UK (London), USA (New Jersey), and Australia (Melbourne) to maximize English language domains as well as the heterogeneity of websites. This procedure returned a total of 1453 unique domains. All domain counts were aggregated, and we computed two popularity metrics per domain: (1) the number of times a domain appears overall for all seeds (absolute frequency), and (2) the number of unique seeds associated with a specific domain (relative frequency). These two metrics were chosen to obtain a large portion of pages (absolute method) and a wide coverage of seeds (relative method). The top 120 domains for each metric were visually inspected to remove potential conspiracy websites (none appeared), less relevant websites such as those not related to text content (YouTube, Amazon, Instagram, Pinterest, LinkedIn, Shutterstock), websites with user-generated content (Blogger, Facebook, Twitter), and other websites such as those related to movie reviews, private companies, and online courses. Following these exclusion criteria, a total of 19 domains were removed. Keeping all domains appearing in both metrics (*N* = 135), this list was visually inspected and subdomains were aggregated (e.g., keith.seas.harvard.edu, sitn.hms.harvard.edu, health.harvard.edu, hsph.harvard.edu aggregated to harvard.edu) while removing mistakenly extracted domains (e.g., www) and non-English domain suffixes (e.g., nationalgeographic.fr). This left us with 93 domains.^6^

### URL extraction and cleaning

Once we had obtained the list of seeds and the two lists of websites, we proceeded with collecting the webpages’ URLs through Google. Besides being the most popular search engine (ranked # 1 worldwide according to www.similarweb.com, accessed September 2020), we used Google Search because we were interested in mimicking user behavior. Importantly, while allowing us to automate URL extraction, this procedure also uses the same search criteria for all websites, without relying on website-specific search engines that might have biased results (e.g., by using the search bar within the website).

URL scraping was performed in R (R Core Team, 2019), using the *curl* package (Ooms, 2019). We formed Google queries by crossing each seed with each website to search for a specific seed within a specific website. For example, the Google query *site:bbc.com moon landing*^7^ returned results about moon landing from the BBC website. The UK top-level domain “google.co.uk” was chosen over “google.com” to ensure English language searches (“.com” in Switzerland—where the study was conducted—automatically returns results in either German, French, or Italian). We also prompted Google to extract results in the English language by adding “hl=en” to the query. For each query, we extracted the first 60 results. Data collection occurred between May 20th and July 4th, 2020 (see workflow in SM2).

Once the URL collection was complete (*N*_conspiracy_ = 67,813; *N*_mainstream_ = 163,488), we proceeded with removing duplicated and non-relevant URLs. This was performed by searching (with regular expressions) and removing the URLs that did not include the website searched, non-text files (pdf, pictures, videos), video and photo galleries, feeds, forums, and blogs, dynamic pages (e.g., URL ending with “php,” “?”), collection pages and archives of links, shops and stores, and Wikipedia lists and discussions. This procedure left us with 29,885 conspiracy and 105,461 mainstream documents.

### Text extraction and cleaning

To extract the HTML files and then the useful text from our list of URLs, we tested several Python packages. These scripts, called “boilerplate stripping,” remove noise text from webpages such as navigation links, header and footer sections, etc. The Python *Goose* package returned the best performance (see SM3) and therefore was chosen for extracting the texts. Importantly, *Goose* can be set to return a series of meta-descriptions and tags from the raw HTML file. Therefore, along with the main body of the text, we used *Goose* to extract the title of the document, the language tag (further capturing non-English pages), and the date the file was uploaded on the website or created (see discussion in “Date” section).

Once all the texts were collected, we further cleaned the raw corpus using the following exclusion criteria: documents for which the HTML meta-tag language was not set as English, empty documents, exact duplicated texts, and texts shorter than 100 words.^8^ In order to further remove non-English documents that did not contain the language HTML tag, we removed texts in which the percentage of top 1000 English words (Fry, 2000) was below 40% (threshold chosen after visual inspection). Finally, we also removed texts whose word count was 2.5 standard deviations above the mean of the whole corpus. This procedure left us with the final LOCO sample of 23,937 conspiracy and 72,806 mainstream documents (see Table 2 for details).

### Lexical feature extraction

For each document in LOCO, we extracted measures of language use with two word-counting tools, namely LIWC (Linguistic Inquiry and Word Count, see Tausczik & Pennebaker, 2010) and Empath (Fast et al., 2016). Both tools have been used previously to investigate the language of conspiracy on social media (Fong et al., 2021; Klein et al., 2019). These tools work on the same principle: they analyze texts, word by word, and check whether the word is included in a predefined category; if so, the category value increases. To extract LIWC categories, we used the LIWC standalone application (version 2015), while for Empath we relied on CLA (Custom List Analyzer version 1.1.1, see Kyle et al., 2015), a standalone application that, along with the batch of texts, takes as input an ad hoc list of dictionaries. Both tools provide standardized outputs, that is, the number of words in a given category divided by the total number of words from the text file. Note that the two tools provide different formats for their output: while LIWC returns percentages (range: 0–100), Empath returns ratios (range: 0–1).

Although these tools work on the same principle, they differ in how they were built, making them somewhat complementary. First, unlike Empath, LIWC detects grammatical categories such as articles, prepositions, pronouns, etc. Second, while LIWC construction relied on human coding, Empath categories were built in a data-driven fashion from a semantic database. For instance, by seeding terms such as “facebook” and “twitter,” Empath generates the category labeled “social media.” The two methods by which these tools were built explain why they compute slightly different values along their categories, as shown in between-dictionary correlations (see Section SM4).

### Topic extraction

For each document in LOCO, we quantify the semantic content by providing a fine-grained topical distribution. This represents a vector containing the probabilities that each of a series of topics is associated with each document. This was achieved with Latent Dirichlet Allocation, (LDA; Blei et al., 2003, see SM5 for text preprocessing). LDA is an unsupervised probabilistic machine learning model capable of identifying co-occurring word patterns and extracting the underlying topic distribution for each text document. By setting a priori the number of topics in a given corpus, LDA computes, for each document in the corpus, the probabilities for all topics of being represented in the document. Meanwhile, each word of the corpus has a probability of being part of a topic. In other words, a word *x* has probability *β* of being part of topic *k*; a topic *k* has probability *γ* of being part of document *n*. The sum of all the word probabilities within one topic is 1, and the sum of all the topic probabilities within one document is 1.

In LDA, the “right” number of topics is determined by the goal of the task more than the data itself (Nguyen et al., 2020, but see also clustering algorithms in general; von Luxburg et al., 2012). LDA topics can be thought as the resolution of a microscope (Barron et al., 2018; Nguyen et al., 2020): if a fine-grained resolution is required, then a large number of topics is better; if the number of topics is small, these topics become more general (Allen & Murdock, 2020). Here, topic extraction was performed with the *topicmodels* R package (Grün & Hornik, 2011), using Gibbs sampling. We left the other LDA parameters set as default, while setting the same seed for reproducibility for all topic extractions. We performed topic extraction with three different levels of resolution, setting *k* at 100, 200, and 300 topics. As a consequence, summing all *k* topics, we obtained 600 topics (see Section SM6 for a thorough description of topics and Section SM7 for topic comparison between different *k*s). In Section SM7.1 of the supplemental material, we have suggested a way to assess topic specificity based on the position of a theme’s keyword (e.g., “Diana” for Princess Diana) within the beta weight-ordered topic’s terms, and the correlation with lexical features. If the theme is event-based (e.g., disappearance of Malaysia Airlines Flight 370 [MH370], 8 March 2014), we also suggest visually inspecting the gamma values plotted over time.

As a proxy for document topic, for each of the three sets of *k* topics, we extracted the topic that had the highest probability of representing the document, i.e., the highest gamma value within all topics within *k*, and included it in the LOCO dataset (see dataset description in “Data availability” section). This means that each document is associated with three topic labels, one for each *k*. We chose this option so as to offer LOCO users a way to perform analyses on a specific topic resolution. Note that we did not provide labels for document topics. Instead, we provide the top 15 words for each topic that, taken together, summarize the topic’s content (Nguyen et al., 2020, and see also beta weight distributions by *k* in Section SM6.1).

We provide with LOCO the matrix containing all gamma values for each document and topic pairs (see “Data availability” section). This results in a matrix with a dimensionality of 96,743 documents × 600 topics. This is useful for obtaining a fine-grained topic description for each document. For example, if a document *n* has the topic with the highest *ɣ* = .90, then this topic has 90% probability of representing document *n*, while the remaining 10% is distributed among all other topics. Similarly, if the highest *ɣ* = .10, all the other topics, by exclusion, occupy the remaining 90% of probabilities. While in the first case we can say that document *n* is well represented by a topic *k* (where gamma is maximum), in the second case, the low gamma value shows that the document *n* is not well represented by a topic *k*. LOCO contains all *ɣ* values, allowing the user to select their own threshold when selecting documents based on topic.

#### Data associated with LOCO’s topics

In order to facilitate topic exploration prior to data analysis, we attach additional files to LOCO that offer an in-depth description of topic content. The first one is a matrix that contains all gamma values for each topic for each document (topic_gamma.json). Because there are three sets of *k* topics (100, 200, and 300), we have named each topic adding the *k* resolution as prefix. For example, the fifth topic of k200 is labeled “k200_5,” while the 134th topic of k300 is labeled “k300_134.” Note that, because we merged the three sets of *k*s into a unique dataset, the sum of topic probabilities for each document is now 3 (1 for each *k* set of topics). The second file (topic_description.json, see also description in SM6) includes descriptions for each of the 600 topics. Descriptions include the top 15 terms ordered by beta weight, the number of documents in which the topic has the highest gamma, the highest correlation with other topics and highest correlation with lexical features (both LIWC and Empath). We also provide a series of plots (in the file “topic_by_time.pdf,” see description in SM6), one for each topic, that track the evolution through time (from 1995 to 2020, see e.g. Fig. 2) of the gamma values. Each plot also includes the topic name and the list of the top 15 terms, ordered by beta values. We believe that these plots, along with the description of each topic (and the actual matrix with gamma values), will help researchers not only in exploring topic associations and lexical features, but also in visually inspecting topics prior to data analysis.

### Representative conspiracy theories

Because one might be interested in what a prototypical conspiratorial language is, we aimed at extracting a set of the most representative CT documents on the basis of the most frequently occurring words within the conspiracy subcorpus. We believe that a set of representative documents may allow researchers to make inferences about CTs more generally. As such, a representative document should share more words with the conspiracy subcorpus compared to a less representative document. Recurrent word patterns such as “they are trying to KILL US!” (from document C01b90) or “know the truth” (document C073a0) might in fact be highly shared across conspiracy documents; hence they would be represented to a larger extent in the conspiracy universe.

Following this reasoning, we extracted the documents that were most similar to the entire conspiracy subcorpus. As a measure of representativeness, we computed the cosine similarity (CS) between words of each document against all words in the conspiracy subcorpus (for a similar procedure, see e.g. de Vries et al., 2018). Text preprocessing was the same as we used to extract LDA topics (see SM5). Documents’ CS was computed using the textstat_simil function from the R package *quanteda*. Values range from 0 to 1, indicating either no overlap (0) or a perfect overlap (1) of terms. This returned a vector for each conspiracy document that indicated the similarity between it and all other conspiracy documents. We averaged this vector to obtain a single value for each document. We finally labeled as “conspiracy representative” the documents whose CS value was higher than one standard deviation above the mean. This resulted in a subset of 4,227 documents, that is, 17.66% of the conspiracy subcorpus. In Section SM8 of the supplemental material, we report the top five documents with the highest and lowest cosine similarity.

### Metadata

#### Mentioning “conspiracy”

We marked documents that mentioned conspiracy in the text. This was done by searching, via regular expressions, and counting the occurrences of the word “conspir*.”^9^ This measure helps keep track of mainstream documents that mention conspiracy which may contaminate mainstream language with details about the corresponding conspiracy (e.g., Pizzagate or Illuminati, themes that rarely appear outside the context of CTs). Therefore, instead of removing these documents, as they represent a special case of mainstream media whose focus is on CTs, we left them in LOCO and annotated the number of instances of the word “conspir*.”^10^ In the conspiracy subcorpus, a total of 3520 documents mentioned conspiracies at least once, while in the mainstream subcorpus there were 5031 documents. On average, conspiracy documents show more instances of “conspir*” than mainstream documents (conspiracy: *M* = 0.351, *SD* = 1.548, range: 0–75; mainstream: *M* = 0.211, *SD* = 1.735, range: 0–182, *t*_(45246)_ = 11.773, *p* < .001, *d* = 0.09). However, when the instances were normalized per word count (i.e., divided by numbers of words in text), there were no differences, *t*_(49107)_ = .993, *p* = .321, *d* = 0.01.

#### Text statistics

For each document, we calculated the number of words, sentences, and paragraphs using the Tool for the Automatic Analysis of Cohesion, TAACO (Crossley et al., 2016, 2019), a freely available standalone application that allows batch processing of text files. Although LIWC also provides measures of word count, which correlates highly with TAACO word count, *r* = .9996, we relied on TAACO measures for two reasons. First, based on the Python Natural Language Toolkit (Bird et al., 2009), TAACO extracts the part of speech for each word, from which it derives a text word count as well as the number of sentences and paragraphs. This, we believe, is a more sophisticated way than merely counting instances of characters separated by spaces. Secondly, because the word-per-sentence measures of LIWC and TAACO correlate poorly, *r* = .59, we visually inspected documents with the highest discrepancy between the two tools. We discovered that LIWC performs poorly when full stop periods are missing from sentences, whereas TAACO considers the new line as a valid sentence-separator marker. Therefore, in LOCO, we keep both LIWC and TAACO word counts, but for consistency with paragraph and sentence counts, we report here (see Table 2) only the TAACO word count.

#### Date

**Fig. 1:**
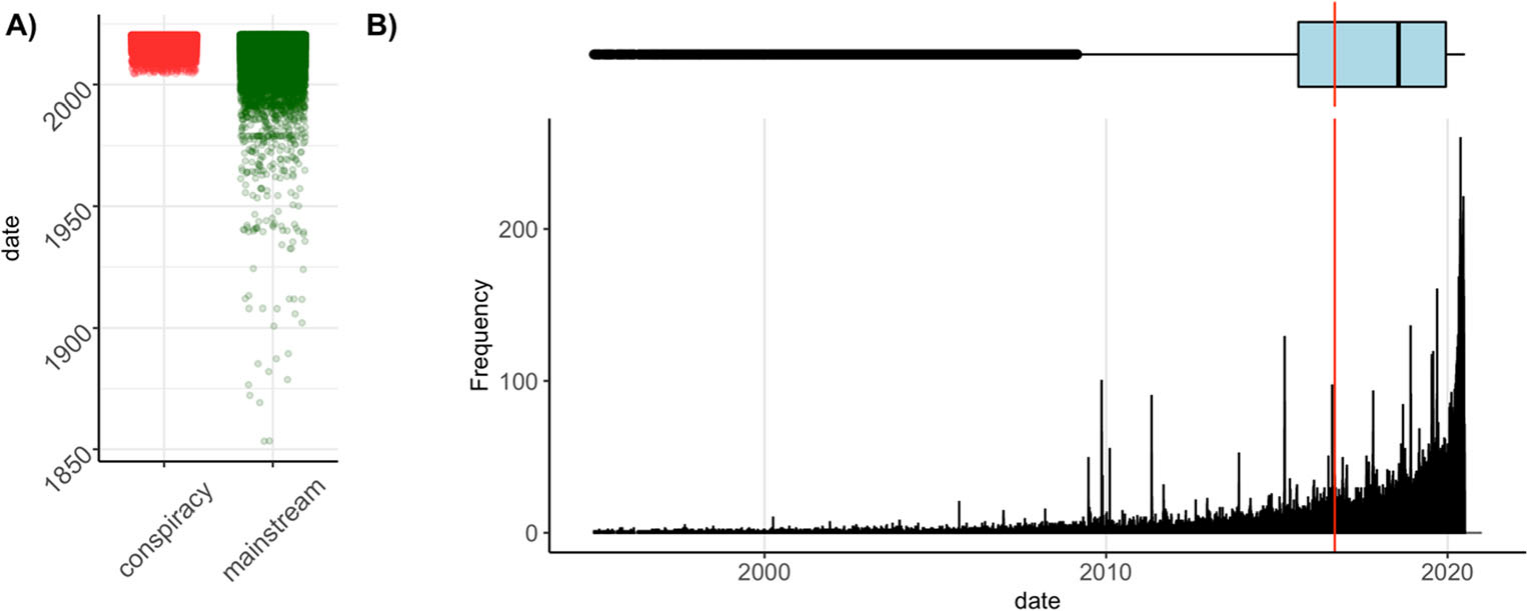
Distribution of documents in LOCO by date*.* Distribution for **a** each subcorpora (red: conspiracy; green: mainstream) and **b** all documents from 1995 to the time of data collection (the red vertical line represents the mean, the boxplot on top displays the median and the interquartile ranges)

Information about document date was obtained primarily from the *Goose* package, which extracts the upload date directly from the raw HTML document. When date was not available (i.e., *Goose* returned an empty cell), we extracted the upload date with regular expressions from the URL of the document (e.g., “http://[…]/2018/01/23/[…].html” was coded as 23 January 2018). In LOCO, date data are provided for 63,868 documents (67% of the entire corpus; 56.67% conspiracy and 69.09% mainstream), see distribution of documents by date in Fig. 1.

**Fig. 2:**
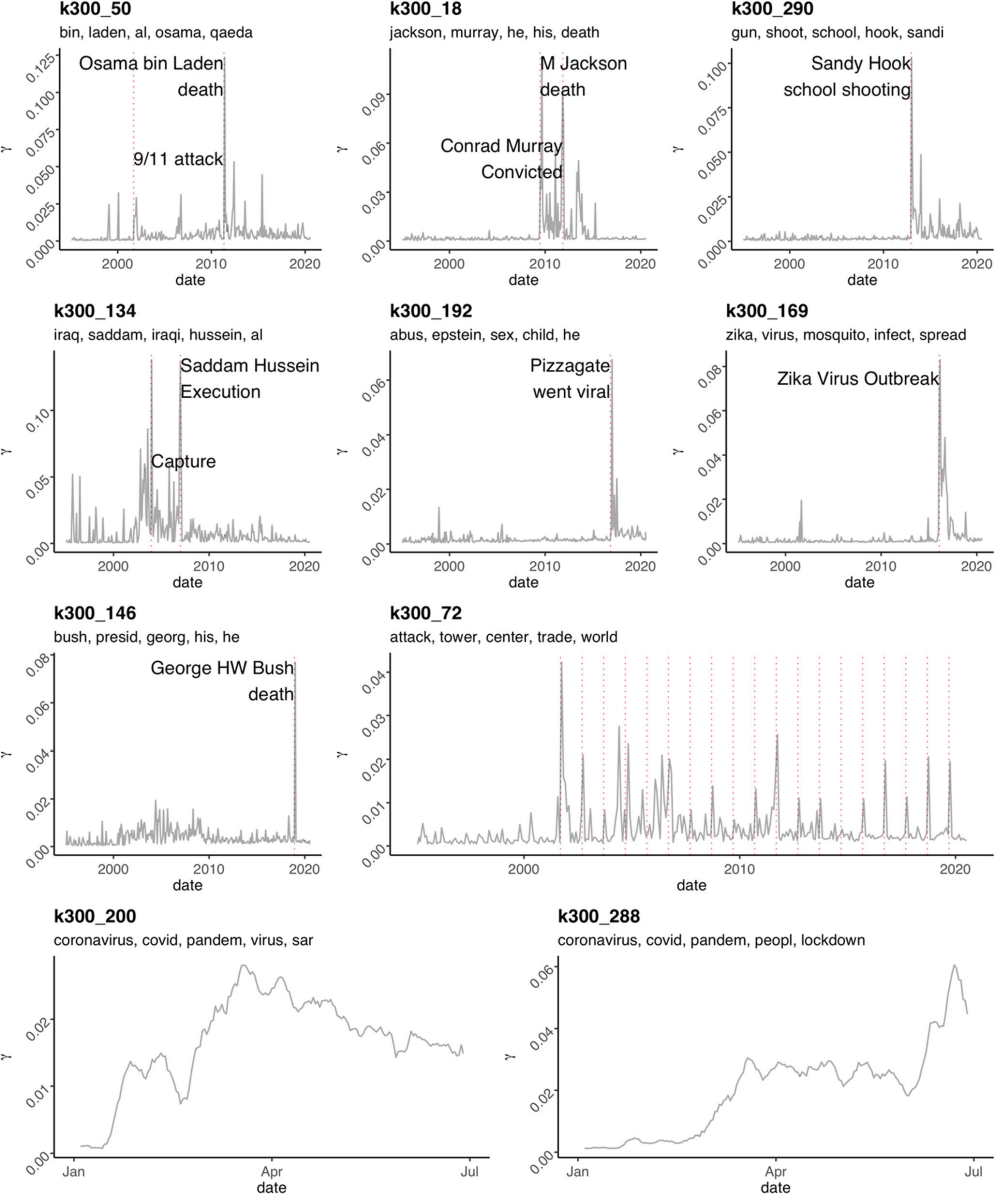
LDA topic gamma values over time**.** The red dotted vertical lines represent the occurrences of significant events associated with the topic. In the 9/11 topic, each vertical line represents September 11th in each year, starting from 2001. Coronavirus topics (bottom) are distributed over the year 2020 (from January to July, when LOCO data collection ended).

It must be noted that date values reflect either the upload date or the authoring date. Both types of information would be informative for different purposes: texts that were authored on the same date are based on a similar level of available information/evidence; texts that were published on the same date compete for audience attention.^11^ While dates before the internet era (e.g., 1853) refer unambiguously to the authoring date, this is less clear for more recent documents. We believe that this information might be nevertheless useful, and therefore we provide all dates available in LOCO. We warn researchers to be aware of date ambiguity before testing any time-related hypothesis. Researchers can either set a threshold for documents’ dates to keep (e.g., after the internet became widespread or another arbitrary cutoff) or develop a method to disentangle the two. However, although documents’ dates may refer to either authoring or upload date, we show in Fig. 2 that documents’ dates are nevertheless linked to the social events discussed in documents.

Lastly, date range differs between mainstream and conspiracy subcorpora, see Table 2. We do not know the reason for this difference, considering that our Google search was independent from documents’ upload date. One possible explanation is that conspiracy websites, being less popular (see Table 5), are also developed with less standardized protocols (see e.g., www.w3c.org). This might have resulted in a less methodical use of HTML meta-tags and therefore the lack of date in some documents. This might also explain the higher percentage of missing dates in conspiracy documents (56.67%). If this is the case, some documents predating 2004 (i.e., the oldest conspiracy document in LOCO) might be in this corpus yet lacking the date. Alternatively (or complementarily), conspiracy websites might be younger, overall, than mainstream websites. For example, the infowars.com domain was registered on 1999-03-07 (data obtained from https://who.is), 911truth.org on 2003-01-14, ahtribune.com (less popular in terms of monthly visits among LOCO’s conspiracy websites) on 2015-08-23, and worldaffairsbrief.com (most popular) on 2004-04-06. In contrast, scientificamerican.com was created on 1997-05-02, sciencemag.org on 1996-04-28, cnn.com on 1993-09-22, and bcc.com on 1989-07-15. Although not tested systematically, those few observations suggest that, overall, conspiracy websites in LOCO might be younger than mainstream ones, therefore explaining the different date ranges.

**Table 5:** Differences between conspiracy and mainstream website metrics

	Mainstream			Conspiracy			*t*-test statistics (raw)			*t*-test statistics (log)		
	*M*	(*SD*)	*N*	*M*	(*SD*)	*N*	*t*	*p*	*d*	*t*	*p*	*d*
Total monthly visits	102,285,513	(191,306,614)	92	965,242	(2,115,315)	28	5.08	***	1.10	14.00	***	3.02
Global rank	7313	(21,765)	89	211,904	(168,890)	28	−6.39	***	1.39	–17.11	***	3.71
Website size	6,844,908	(16,049,205)	92	6224	(12,918)	58	4.09	***	0.69	18.43	***	3.09
FB projected shares	3,213,458,353	(9,348,074,961)	92	27,11,190	(14,540,274)	58	3.29	**	0.55	12.78	***	2.14
Traffic, direct^†^	28.95	(13.45)	92	57.55	(21.57)	28	–6.63	***	1.43			
Traffic, search^†^	56.83	(17.49)	92	13.82	(10.44)	28	16.00	***	3.45			
Traffic, social^†^	8.08	(5.58)	92	18.4	(19.28)	28	–2.8	**	0.60			

*Note*. Differences tested with Welch's unequal variances *t*-test. Log transformation was applied to highly skewed variables after having added a constant 1 to avoid -Infinite values when the raw score was zero. ^†^Values expressed as percentages and not log-transformed. *d*: Cohen’s *d*. FB: Facebook. Website size is expressed in number of webpages

#### Facebook shares

For each webpage, we obtained information about spread from the web tool sharedcount.com (SC). Via an application programming interface, SC retrieves from Facebook^12^ the number of shares, comments, and reactions for each webpage URL. According to the website, SC reports “all time statistics,” which means that values refer to the overall shares since the creation of the URL tracked. All data from SC were collected in September 2020.

Besides single URL shares, we also computed an estimation of the total number of shares from the observed data we collected for each website. To this end, we computed the sum of all webpage Facebook shares for each website and divided them by the proportion of sampled LOCO webpage for each website. For instance, in LOCO, there are 967 documents extracted from the website www.infowars.com. Infowars has 15,500 webpages indexed on Google (see “Website metrics” section), which means that LOCO contains 6.24% of all Infowars webpages. The aggregated total Facebook shares of all 967 Infowars documents in LOCO is 89,639. By dividing the total shares (89,639) for the proportion of LOCO documents (0.0624), we obtain an estimation of total website shares, which in this case is 1,436,820 times, a rough estimation of the grand total of shares of all Infowars webpages. Once this measure was computed for all websites, we then tested the correlations of this measure with other spread measures. The estimated Facebook shares correlates with website global rank (*r* = -.81) and with website monthly visits (*r* = .81, see SM9 for more details).

#### Website category

We relied on MBFC for obtaining metrics of political side and factual reporting for each website. MBFC contains manual annotations and bias analyses for over 2,000—mostly news—websites. According to the MBFC method,^13^ each website’s bias is evaluated on four criteria, including biased wording headlines (e.g., the source uses loaded words to convey emotion to sway the reader), factual sourcing (e.g., the source reports factually and backs up claims with well-sourced evidence), story choices (e.g., the source reports news from both sides), and political affiliation (e.g., the source endorses a particular political ideology). Factual reporting is based on the factual sourcing used for assessing bias. For each website, a minimum of 10 headlines and 5 news stories are assessed by MBFC experts. Low and very low factual reporting sources are those that need to be fact-checked for intentional fake news, conspiracy, and propaganda. Although MBFC states that their methodology has been not tested scientifically, they nevertheless adhere to the International Fact-Checking Network fact-checkers’ Code of Principles^14^ and strive for transparency. Furthermore, MBFC annotations have been used by other researchers to study fake news and conspiracy websites (Baly et al., 2018; Cinelli et al., 2021; Pennycook & Rand, 2019; Risius et al., 2019).

For each of the LOCO websites that was reviewed in MBFC, we extracted measures of political orientation (left, left center, least biased, right center, and right), factual reporting (from “very low” to “very high”), pseudoscience level (provided by MBFC only for conspiracy websites), and whether the website was labeled as pro-science (i.e., relying on legitimate science or evidence based on credible scientific sourcing). Note that pro-science websites do not have political orientation labels. Data from MBFC were collected in July 2020.

#### Website metrics

We have extracted a series of website metrics that, overall, offer an idea of popularity, engagement, and size for each website. From the web tool similarweb.com^15^ (SW), we collected data about monthly total visits, global rank, and category. We also collected information about the type of incoming traffic. Expressed in percentage, these metrics partition each website's incoming traffic into direct (when a user reaches the website directly by typing the URL on the web browser or recalling it from bookmarks), from a search engine (when a website is reached through a search engine, e.g., Google), and from social media (when a website is reached through social media, e.g., a post on Facebook or Twitter). Other types of incoming traffic offered by SW, which we did not collect, are referrals, mail, and display, which overall account for about 7% (SD = 6.38) of remaining incoming traffic in our dataset (computed by summing direct, search engine, and social media traffic and subtracting it from 100).

SW was chosen over Alexa.com (a web tool that provides similar services), mainly because SW updates its statistics every month, whereas Alexa provides daily updates. While the latter appears to be more fine-grained, it nevertheless poses some limitations in terms of data collection (which manually spans several days) due to daily statistical fluctuations. In addition, SW offers a wide range of free features, otherwise accessible in Alexa upon a monthly subscription, and, importantly, the SW database is composed of ~50 million websites (vs. ~30 million websites in Alexa). These data were collected in July 2020.

In addition, in order to obtain an estimation of the website size, we extracted the total number of webpages per website indexed by Google. This was done by querying Google with “*site:*” followed by the website.^16^ This data was collected in March 2021.

### Data availability

LOCO’s data is freely available at https://osf.io/snpcg and includes:

1. **LOCO.json** (587.6 MB): a JSON (JavaScript Object Notation) file containing the LOCO corpus itself. 96,746 rows (documents) × 20 columns (see Table 6)
2. **website_metadata.json** (55.3 KB): a JSON file containing websites’ metadata. 150 rows (websites) × 18 columns (see Table 7)
3. **LOCO_LFs.json** (573.1 MB): a JSON file containing the full set of lexical features. 96,746 rows (documents) × 288 columns (*N*_Empath_ = 194; *N*_LIWC_ = 93)
4. **topic_gamma.json** (963.7 MB): a JSON file containing topics’ gamma values. 96,746 rows (documents) × 600 columns (topics)
5. **topic_by_time.pdf** (169.6 MB): a PDF file containing plots of topics’ gamma values over time (from 1995 to 2020). It contains 600 pages.
6. **topic_description.json** (188.2 KB): a JSON file containing detailed descriptions of topics. 600 rows (topics) × 12 columns (see SM6)

**Table 6:** LOCO dataset variables description

Variable name (% empty/missing values, if any)	Variable description
doc_id	Six-character hexadecimal sequence of document unique identification number. The first character stores the source: C stands for conspiracy (e.g., C0004d) and M stands for mainstream (e.g., M095eb)
URL	URL associated with the document
Website	The website from which the document was extracted
seeds (2.26%)	The seeds we used to gather documents. The page was returned by all the keywords listed in this variable (*N* = 47)
date (33.98%)	The date the webpage was uploaded or uploaded (format: YYYY-MM-DD)
subcorpus	Either conspiracy or mainstream (*N*_conspiracy_ = 23,937; *N*_mainstream_ = 72,806).
title (0.11%)	Title of the document
txt	Document text (see text statistics in Table 2)
txt_nwords	Number of words
txt_nsentences	Number of sentences
txt_nparagraphs	Number of paragraphs
topic_k100	The topic ID with highest gamma value within k100 LDA (*N* = 100 unique, e.g., k100_24)
topic_k200	The topic ID with highest gamma value within k200 LDA (*N* = 200 unique, e.g., k200_75)
topic_k300	The topic ID with highest gamma value within k300 LDA (*N* = 300 unique, e.g., k300_192)
mention_conspiracy	Occurrences count for the word “conspir*” in text, see “Mentioning conspiracy” ” section
conspiracy_representative	Logical. TRUE (*N* = 4227) if the conspiracy document is representative
cosine_similarity	Cosine similarity values for conspiracy documents (values > mean + 1 SD are considered representative)
FB_shares (0.01%)	URL’s Facebook shares
FB_comments (0.01%)	URL’s Facebook comments
FB_reactions (0.01%)	URL’s Facebook reactions

*Note*. Percentages of empty/missing values are calculated on the list of documents (*N* = 96,743)

**Table 7:** LOCO’s website metadata variables description

Variable name (% empty/missing values, if any)	Variable description
Website	Website name (*N* = 150)
URL	URL associated with the website domain
n_webpages	Overall number of webpages in website obtained by Google search (see “Website metrics” section)
MBFC_political_orientation (69%)	Political orientation. Left (*N* = 4), left_center (*N* = 19), least_biased (*N* = 15), right_center (*N* = 4), right (*N* = 5)
MBFC_factual_reporting (21%)	Factual reporting. Very_low (*N* = 10), low (*N* = 43), mixed (*N* = 16), mostly_factual (*N* = 4), high (*N* = 35), very_high (*N* = 11)
MBFC_conspiracy	Logical. If TRUE (*N* = 58), website is conspiracy
MBFC_pseudoscience (62%)	For conspiracy websites only. Zero (*N* = 1), mild (*N* = 2), moderate (*N* = 9), strong (*N* = 16), quackery (*N* = 29)
MBFC_proscience	Logical. TRUE (*N* = 16) if website is labeled as pro-science
SW_total_visits (20%)	Total visits, desktop and mobile web aggregated
SW_global_rank (22%)	Traffic rank of website, as compared to all other websites in the world
SW_Category (20%)	Website category (e.g., news_and_media, *N* = 60; health, *N* = 16, science_and_education, *N* = 13)
SW_traffic_direct (20%)	Percentage of direct desktop incoming traffic (from typing the URL in a browser)
SW_traffic_search (20%)	Percentage of search desktop incoming traffic (from a search engine)
SW_traffic_social (20%)	Percentage of direct desktop incoming traffic (from a URL on social media)
FB_shares_homepage	Facebook shares of homepage (see discussion in SM9)
FB_shares_estimated	Estimated overall Facebook shares given total number of website’s webpages (see “Facebook shares” section)

*Note*. Percentages of empty/missing values are calculated on the list of websites (*N* = 150)

## Exploring LOCO’s features

In this section, we explore LOCO’s features and provide examples on how to handle LOCO’s variables and subset corpus. Some of these analyses are descriptive in nature and offer a way to visually explore to what extent LOCO’s data relate to the external world, such as visualizing the evolution of LDA topics through time (see “Topic analyses” section) or exploring to what extent the language used in LOCO’s documents overlaps with the language used in social media (see “Towards corpora of CT texts” section). Other analyses are more explorative, such as testing whether mentioning conspiracy in mainstream documents affects lexical features (see “Effect of mentioning conspiracy” section) or whether conspiracy-representative documents are in fact different from other conspiracy documents in terms of lexical features and spread (i.e., Facebook shares, see “Properties of representative conspiracy documents” section). Lastly, we also explore to what extent LOCO’s higher-level metadata might provide insights into psychological processes by analyzing the behavior of websites’ users (see “Website incoming traffic” section). Overall, these analyses not only suggest how to use LOCO, but also offer insights on the language of conspiracy and the psychology of conspiracy websites users.

### Topic analyses

Each document in LOCO is associated with a vector that encapsulates and quantifies the semantic content, namely the LDA topics. While in the main dataset (LOCO.json) we provide for each document only the label of the most prevalent topic (one for each level of topic resolution, that is *k =* 100, *k =* 200, and *k =* 300), in a separate dataset (topic_gamma.json) each document is associated with the gamma values for all 600 LDA topics extracted. In this section, we explore how LDA topics reflects real-world events by visually inspecting how these LDA topics develop through time for documents whose date was recorded. This reasoning is supported by the fact that, because texts are capable of showing cultural patterns (Lansdall-Welfare et al., 2017; Li et al., 2019, 2020; Michel et al., 2011), a significant social event should be reflected in the texts’ topic time series. To explore this possibility, we selected a set of topics that are associated with a specific event (instead of non-event-specific topics such as AIDS or Illuminati) such as the death of societally significant people: Osama bin Laden (2011-05-02, topic k300_50), Michael Jackson (2009-06-25, k300_18), George H.W. Bush (2018-11-30, k300_146), and Saddam Hussein (2006-12-30, k300_134); outbreaks of pandemics such as Zika virus (2016-02-01, k300_169) and coronavirus (2020-03-11, k300_200 and k300_288); and other significant societal events such as the 9/11 terroristic attack (2001-09-11, k300_72), the Sandy Hook school shooting (2012-12-14, k300_290), and Pizzagate (2016-11-01, when it went viral, k300_192). In Fig. 2, for all documents in LOCO provided with upload/creation data, topic patterns (i.e., gamma values on the *Y* axis) are shown within a time span of 25 years, from 1995 to 2020 (first three rows) and for 2020 (fourth row for coronavirus-related topics) from January to July, when LOCO’s data collection ended.

### Lexical features

#### Overlap with Reddit users’ language

Ideally, a corpus must be representative and replicable, meaning that the sampled data should represent the full range of variability of the population from which the sample is drawn. If our corpus successfully represents CTs, then its content should mirror the content of comments and threads posted by conspiracy believers on social media. To this aim, we compared the lexical features extracted from LOCO’s documents (LOCO_LFs.json) with those extracted from comments on Reddit by Klein et al. (2019). Although user discussions on conspiracy forums are not conspiracy per se, we expect a certain overlap in language features with LOCO documents. This is because, while forums do not offer adequate space to fully develop argumentative discourses, a conspiracy believer can nevertheless express a conspiratorial worldview through language use (e.g., deception: “*They are hiding the cure from us for their own profit!!*”), even in discussion not related to conspiracy. In fact, Klein et al. (2019) compared language features of a group of users who posted in the r/conspiracy subreddit with those from a carefully matched control group of users who never posted in r/conspiracy. Although we do not know to what extent users who posted in the r/conspiracy subreddit endorse CTs, Klein and colleagues found language differences associated with a conspiratorial mindset (e.g., power, deception, dominance) that sees hidden powerful and malevolent enemies at work.

**Fig. 3:**
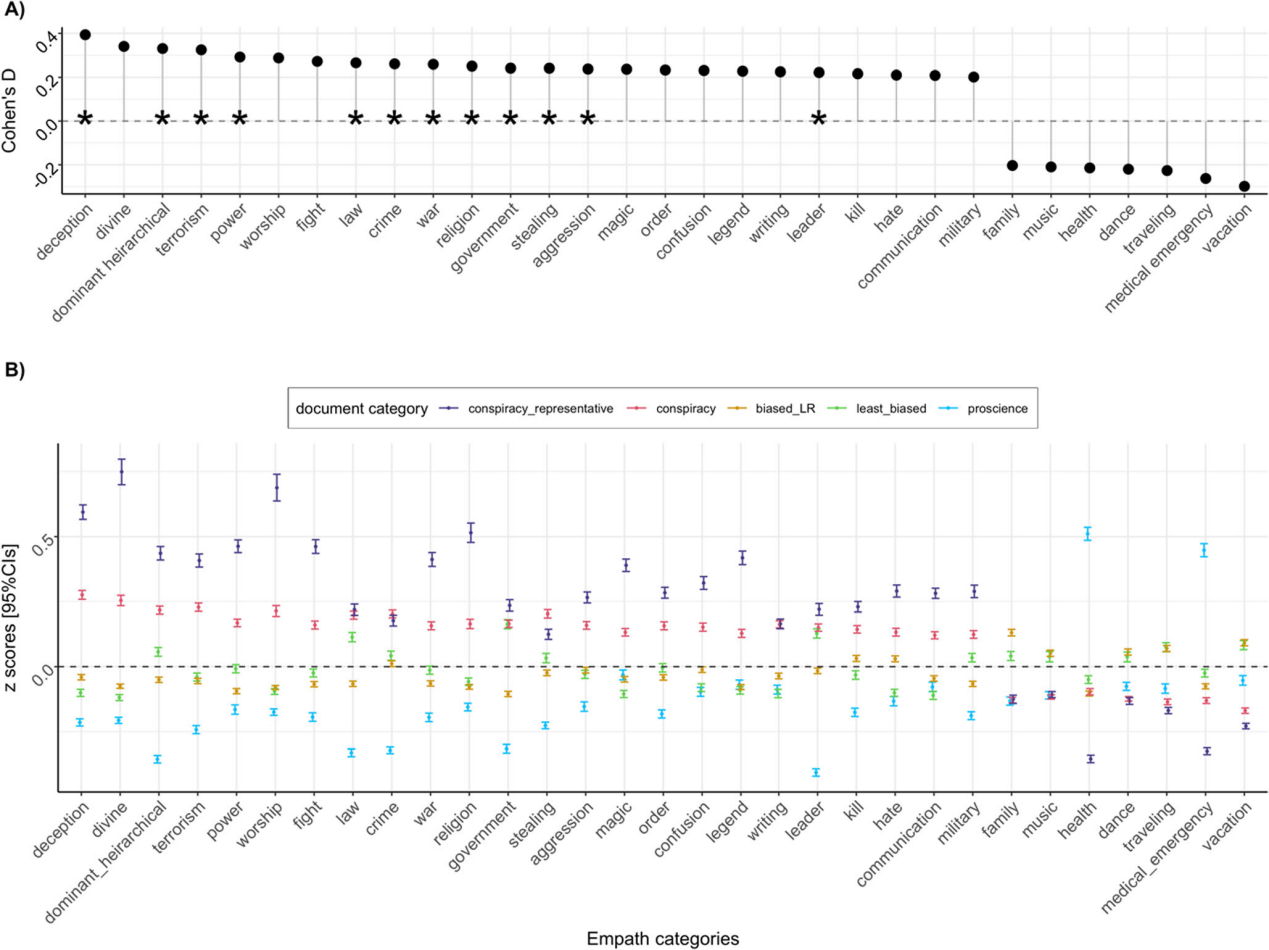
Differences in lexical features between conspiracy and mainstream documents **a** Effect sizes that yielded a Cohen’s *d* > .20 from *t*-tests between conspiracy and mainstream documents on Empath lexical categories. Positive effect sizes indicate that the category value is higher in conspiracy documents. A star indicates that the category emerged as having *d* > .20 also in Klein et al. (2019). **b** Comparison of means [and 95% CIs] for the same set of variables (scaled to *z* values) across different document categories

We proceeded with replicating the method of Klein et al. (2019) on LOCO by comparing our two subcorpora and explored whether the same patterns emerged. Similar to their work, we used the lexical features derived from Empath and tested differences between conspiracy and mainstream documents on the 194 Empath categories. Then, we used Welch’s *t*-test and computed Cohen’s *d* for each test on the variables that yielded a significant difference at *p* < .00026 (Bonferroni correction for 194 tests). Note that here we are not testing any particular hypothesis, but provide this as exploratory analysis to guide future research. Results are shown in Fig. 3. On the top (A), only variables that produced an effect size of *d* > .20 are displayed, arranged in decreasing order. On the bottom (B), each variable was scaled to *z* values, and mean values are shown for different website categories: conspiracy_representative (*N* = 4,227), other conspiracy (*N* = 19,710), biased_LR (aggregating documents biased towards either the left or right, *N* = 31,928), least-biased (*N* = 14,180), and pro-science (*N* = 11,440).^17^

Lexical differences between LOCO conspiracy and mainstream documents overlap with those between Reddit groups found by Klein and collaborators (Fig. 3a). Among the lexical categories characterizing conspiracy language (i.e., positive values in Fig. 3a), half of them emerged as overlapping between the two datasets. In LOCO, other lexical categories were higher in conspiracy (vs. mainstream), such as *divine* and *worship* that correlate with *religion* (*r* = .92, *r* = .95, respectively, in our dataset) found in Klein, and *kill* and hate that correlate with *death* (*r* = .72, *r* = .44) and *negative_emotion* (*r* = .71; *r* = .76) found in Klein but not in LOCO. It is also worth noting that representative conspiracy documents, on average, display an exaggeration of the “average” conspiratorial language as exemplified from the means further departing from zero (this will be further explored in “Properties of representative conspiracy documents” section).

#### Effect of mentioning conspiracy

We explored the possibility that mentioning conspiracy in the text would increase conspiratorial language (see “Mentioning “conspiracy” section). To this aim, we run multiple *t*-tests using as dependent variables the 31 lexical categories that yielded an effect size *d* > .20 from the previous section (see “Overlap with Reddit users’” section). In doing so, we used two different LOCO subcorpora: one (raw) which is based on the whole mainstream data (*N*_mainstream_ = 72,806) and one (cleaned) from which we removed all mainstream documents containing at least one instance of the word “conspir*” (Final *N*_mainstream_ = 67,775). Note that we removed mentions of conspiracy only in the mainstream documents because we aimed to test the difference between the subcorpora removing potential conspiratorial language from mainstream documents, so as to obtain a mainstream subcorpus cleaned of conspiratorial language. We reasoned that conspiracy documents deliver conspiracy language even without mentioning the word “conspir*.” From each test, we extracted the effect size (Cohen’s *d*) and then compared the changes in *d*, with a paired *t*-test, from the raw to the cleaned dataset. Results show an overall increase in the effect sizes, *t*_(30)_ = 5.08, *p* < .001, suggesting that cleaning the mainstream subcorpus from documents that mention conspiracies amplifies differences in language features between the two subcorpora (see SM10 for details).

We finally explored whether mentioning conspiracy had an effect on lexical features. To this aim, we extracted the top four Empath categories that in the previous analysis (see paragraph above) had yielded the largest changes in effect size, namely *crime*, *terrorism*, *deception*, and *stealing*, and tested the correlation (on log-transformed variables, but see SM10 for non-log-transformed results) between the number of mentions of conspiracy and lexical variables. The results showed a positive relationship: crime: *r* = .31; terrorism: *r* = .33; deception: *r* = .21; and stealing: *r* = .13. Overall, these tests show that mentioning conspiracy, even in mainstream documents, affects language features. Therefore, we suggest that researchers carefully evaluate whether or not to include mainstream documents containing the word “conspir*” in their analyses.

#### Properties of representative conspiracy documents

**Fig. 4:**
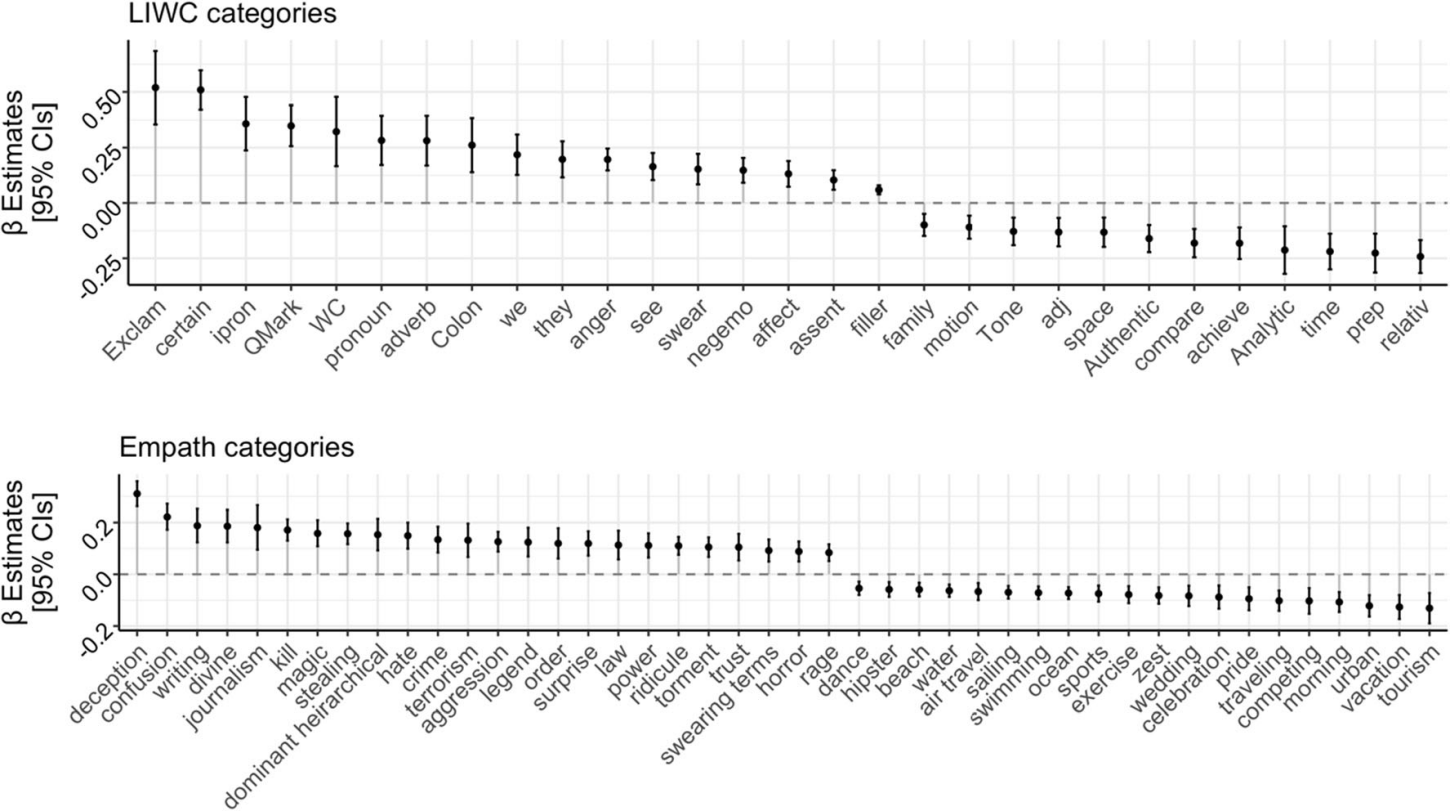
Differences in lexical features between high- and low-representative conspiracy documents. Positive *β* estimates indicate that the category is higher among conspiracy documents that are more representative of the conspiracy corpus as measured by their document cosine similarity with other conspiracy documents in the corpus

We explored to what extent the representative set of conspiracy documents (*N* = 4,227) differs from the other conspiracy documents (*N* = 19,710) in terms of lexical features. To this aim, after subsetting LOCO to only conspiracy documents, we run a series of linear mixed-effects models using the *lme4* (Bates et al., 2015) and the *lmerTest* (Kuznetsova et al., 2017) R packages. In each model, we specified as dependent variables the LIWC (*N* = 93) and Empath (*N* = 194) categories, and as fixed effects the dichotomous representativeness predictor. As random intercept, we specified both the websites from which documents were extracted and the topic label with the highest gamma value for *k* = 100 because, being less specific, it provides a more inclusive clustering that aggregates similar topics. In other words, while for *k* = 300 we would have had several LDA topics revolving around a theme, with a lower topic resolution, topics are more general (we replicated the same analyses with *k* = 200 topics, and results are not visibly different, see SM11). Before entering the model, the dependent variables were scaled to *z* values. Standardized *β* estimates are displayed in Fig. 4 for only the dependent variables that were significant at *p* < .00017 (Bonferroni correction for 287 tests) by the dichotomous predictor. Positive estimates indicate that the category is higher in the representative subset of conspiracy documents.

The representative conspiracy subset is generally more emotionally charged than the other documents, as displayed by the higher value for the category related to affective processes (LIWC category *affect*), and more specifically to negative emotions (LIWC categories *anger*, *swear*, *negemo*). Representative conspiracy documents, as compared with the non-representative conspiracy documents, display a prototypical language of conspiracy focused on power, dominance, and aggression (Empath categories *deception*, *dominant hierarchical*, *kill*, *hate*, *order*, *power*, *aggression*, and *rage*).

As for the rhetorical style used by the representative subset, we observe higher values for certainty (category *certain*), and interrogative (category *interrog*) language, along with higher use of question and exclamation marks (categories *Exclam*, *QMark*). This is in line with the observation that the rhetorical style of conspiracy narratives is built upon refutational strategies based on questioning the dubious version of the official story while highlighting the lack of answers from official sources (Oswald, 2016).

In line with research on social motives underlying belief in CTs (Douglas et al., 2019), the higher use of *we* and *they*, along with affiliative (LIWC category *affiliation*) and social (category *social*) language, suggests a process of social identification of the ingroup (*we*) by exclusion from the outgroup (*they*).

Overall, as already seen in Fig. 3 and in the work of Klein et al. (2019), the representative conspiracy documents seem to be an exaggerated version of an average conspiracy document, characterized by language of power, action, and dominance. They are at the same time less likely to display non-conspiratorial language as exemplified by lower values for categories such as *tourism*, *vacation*, *urban*, and *morning*. Interestingly, these patterns overlap with those found on Twitter, in which lexical differences between conspiracy and science influencers were identified in the use of negative emotion (e.g., anger) and a focus on topics such as death, religion, and power (Fong et al., 2021).

If the representative documents are rhetorically appealing and emotionally loaded, then we can expect that they will spread more successfully than the other, less representative documents. This reasoning is also in line with the fact that emotional content is a successful feature of narrative stickiness and transmission (Franks et al., 2013; Heath et al., 2001). Therefore, we tested whether the representative subset of conspiracy documents spread more than non-representative conspiracy documents. To this aim, we fit a linear mixed-effects model predicting Facebook shares (log-transformed). We set conspiracy representativeness as predictor along with website total visits as covariate while specifying a random intercept for websites. Results showed that the subset of representative conspiracy documents was more shared on Facebook compared to the other conspiracy documents, *β* = 0.121, *SE* = 0.017, *t* = 7.075, *p* < .001.

### Website incoming traffic

Besides the texts themselves and the documents and their metadata, LOCO is also provided with higher-level metadata, namely information about its constituent websites. Such a set of variables (contained in the file website_metadata.json) might be useful for testing hypotheses at the website level. For example, here, we describe the behavior of conspiracy and non-conspiracy communities from websites traffic information.

Analysis of online social media shows that users tend to aggregate in echo chambers that are homogeneous clusters of communities of interest (Bessi, Coletto, et al., 2015a; Brugnoli et al., 2019; Del Vicario, Bessi, et al., 2016a). Such clustering is reinforced in online and offline social networks (Del Vicario et al., 2017) whereby a like-minded trusted node in the social network (a friend or a page followed on Facebook) shares content that adheres to a system of beliefs. Moreover, within online social networks, users access information through a narrower spectrum of sources compared to web searches (Nikolov et al., 2015), meaning that being embedded within a social bubble reduces exposure to different viewpoints. When users of conspiracy Facebook pages are exposed to debunking information, they increase traffic towards conspiracy-like content (Zollo et al., 2017). This behavior suggests a confirmation bias: people avoid cognitive dissonance while searching for reinforcement (Brugnoli et al., 2019; Hills, 2019).

Website incoming traffic provides similar information about user behavior. For example, direct traffic may indicate a certain level of loyalty or at least that the user knows the website or has learned about it through their social contacts (Pauwels et al., 2016). When a website is reached from a search engine, the website is not necessarily known to the user. Put differently, how people arrive at a website may indirectly reveal information about their prior knowledge, beliefs, and social community. If echo chambers provide links to belief confirming content, then a confirmation bias theory of conspiratorial thinking would predict that users of conspiratorial websites are more likely to arrive there via a bookmarked URL or through online social networks than through impartial search engines.

To explore this possibility, we analyze user behavior through website incoming traffic (see “Website metrics” section). Because of a link between confirmation bias and belief in CTs (Del Vicario et al., 2017; Del Vicario, Bessi, et al., 2016a; Marchlewska et al., 2018; Meppelink et al., 2019; Zollo et al., 2017), we expect that conspiracy websites display higher levels of direct traffic and lower levels of search traffic. Conspiracy ideas spread within homogeneous social media communities of like-minded believers who share conspiracy narratives; thus we also expect that traffic from social media (i.e., incoming traffic from a social media link) is higher in conspiracy compared to non-conspiracy websites. Moreover, because of known links between partisanship polarization and echo chambers (Stroud, 2010), confirmation bias (Westen et al., 2006), and belief in CTs (van Prooijen et al., 2015) we explored whether politically polarized websites (on both left and right sides of the spectrum) show patterns comparable to those of conspiracy websites compared to least biased websites.

**Fig. 5:**
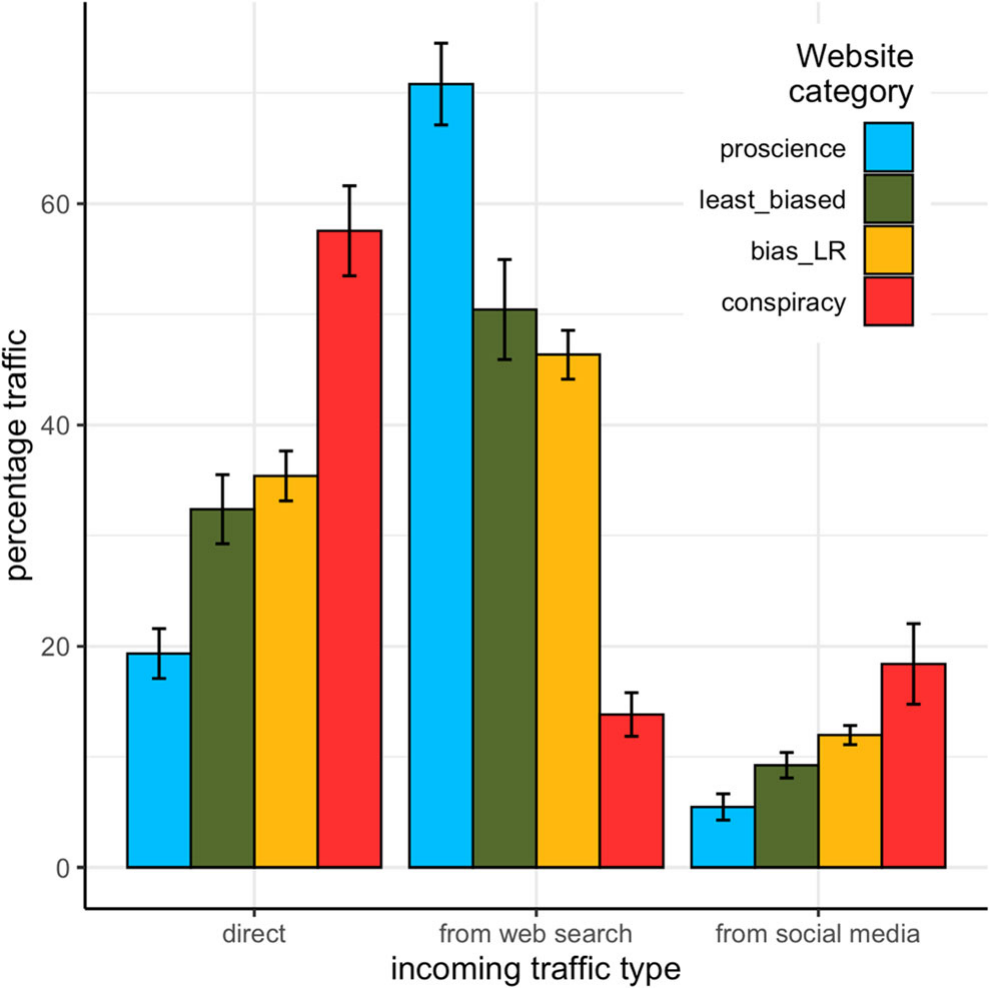
Types of incoming traffic by website category*.* Average of websites’ percentages of incoming traffic (direct, from web search, and from social media) by website categories. Error bars represent the standard error of the mean

We selected the websites (for which traffic data were collected) labeled as conspiracy (*N* = 28), least biased (*N* = 15), and pro-science (*N* = 16), and aggregated the websites leaning on either the left or right of the political spectrum, labeling them “biased_LR” (*N* = 32). Analysis of variance and post hoc comparisons using Tukey’s honestly significant difference (HSD) test were used to test differences in traffic type between website categories. Direct traffic was highest for conspiracy (*M* = 57.55, *SD* = 21.57) and lowest for pro-science (*M* = 13.35, *SD* = 12.12), *F*_(3,87)_ = 23.41, *p* < .001. All post hoc differences among the four categories were significant at *p* < .01 except differences between least biased and biased websites (*p* = .92) and between pro-science and least biased websites (*p* = .09). As for traffic from search engines, the highest rate was on pro-science websites (*M* = 70.80, *SD* = 14.80) and the lower on conspiracy ones (*M* = 13.82, *SD* = 10.44), *F*_(3,87)_ = 70.46, *p* < .001. All differences were significant (*p*s < .001) except those between least biased and biased websites (p = .76). Incoming traffic from social media sites was higher in conspiracy (*M* = 18.40, *SD* = 19.28) than in pro-science (*M* = 5.44, *SD* = 4.76), *F*_(3,87)_ = 4.93, *p* < .01; all other differences were nonsignificant. Results are shown in Fig. 5.

These results suggest that CT websites are predominantly reached by the users typing the URL on their browser (or by recalling the URL from bookmarks) or following a link posted on social media. On the contrary, pro-science websites are mostly accessed from web searches. Differences in access routes between biased and least-biased websites were not significant. This indicates that though users of conspiracy websites are most similar to users of biased websites, they are nonetheless in a category of their own.

## Discussion and conclusions

LOCO is a multilevel, richly annotated, topic-matched corpus of CTs composed of nearly 100,000 documents, with a total of 88 million words. This represents a rich source of data for better understanding the content and spread of CTs. LOCO is also freely available (https://osf.io/snpcg). Being for the most part composed of texts with additional metadata and lexical features, LOCO is conceptualized as a turnkey resource from which researchers can test hypotheses, further extract features, and/or build classification and predictive models. To this end, while building LOCO, we aimed at obtaining a large yet representative set of documents that also provided a set of metadata that could be used ad hoc to partition LOCO prior to analysis.

A large portion of the present paper has focused on thoroughly describing the methodology on which LOCO was built. As we have built LOCO upon previous works’ strengths and weaknesses, we believe that a meticulous method description will also allow future research to benefit from LOCO’s strengths and weaknesses, opening up possibilities for further data collection in the field of CT studies.

Our analysis of LOCO demonstrates its potential by making a number of contributions to the conspiracy research literature: (1) By mapping topics on document dates, we show that LOCO’s documents track important social events. (2) We replicated the lexical analysis of previous work, finding an overlap between LOCO documents and comments on online social media. (3) We find that mainstream documents that mention conspiracy display conspiratorial language. (4) We have extracted and analyzed the language of prototypical conspiracy documents and find that these amplify features of conspiratorial language. (5) We find a pattern of website traffic indicating active online social media communities and the potential for confirmation bias via direct traffic. And (6) we find that conspiracy websites show statistically different patterns of web traffic than biased (politically left or right) websites, suggestive of a difference in their users. At the same time, we have provided suggestions on how to use LOCO to make new contributions.

Because we relied on a multitude of heterogeneous methods, we also believe that each of our corpus construction stages can benefit data collection for text analysis research in general. While we built LOCO on a specific narrative genre, namely CTs, the same methodology, or part of it, can be employed for other purposes. For example, researchers may be interested in comparing a list of websites against another one, or comparing webpages returned by specific sets of seeds, or, as we have done, do both at the same time by crossing lists of websites and seeds. We have also shown that it is possible to rely on several tools to enrich a web-based set of text with meta-data, such as political biases and fact accuracy (from MBFC), measures of spread (from SC), and popularity and traffic (from SW). Other freely available tools we have employed are available for text extraction (*Goose*) and analyses such as Empath, TAACO, as well as the *quanteda* and the *topicmodels* packages.

Because we also provided the URLs associated with each document, it is potentially possible to extract HTML data in order to analyze web markup features as previous work has done on fake news (Castelo et al., 2019). Moreover, different sets of psycholinguistic measures can be extracted from LOCO’s texts, such as word norms for valence, arousal, and dominance (Warriner et al., 2013), imageability (Cortese & Fugett, 2004), frequency (Brysbaert & New, 2009), concreteness (Brysbaert et al., 2014), and age of acquisition (Kuperman et al., 2012).

In conclusion, LOCO is a rich source that helps to better understand the content of CTs. Here, we have explored how CT users behave online and which language features are associated with the spread of documents over social media, and we sketched a preliminary overview of the lexical fingerprint of the (prototypical) conspiratorial language. Therefore, LOCO’s contribution is multiple: while providing data mainly for lexical analysis and document spread, it can also help to reveal psychological processes. For the sake of global public interest, given the detrimental potential consequences associated with the endorsement of CTs, understanding how CTs spread is critical to ultimately limiting their negative consequences.

## Supplementary Information

ESM 1 (PDF 7022 kb)

## Footnotes

1 The acronym LOCO might suggest the idea that conspiracy theories and theorists are all crazy. Far from this position, we rather highlight the polarizing phenomenon by which, regardless of the belief position, the “others” are considered crazy. People with beliefs in CTs feel, and often are, stigmatized (Lantian et al., 2018). On the other hand, nonbelievers are also in some instances mocked as “globetards,” “vaxholes,” or “covidiots.” The last expression is emblematic as it is used by both sides of the belief spectrum to refer to people who either believe or do not believe in COVID-19. And, of course, some conspiracy theories are true.

2 Note that we make a distinction between documents, webpages, and websites’ metadata. For document, we refer to the text and its intrinsic features such as title, topic, lexical features, etc. Differently, for webpage, we refer to a set of paratextual information related to the webpage (that contains the text) such as the URL, date, spread, and the website host. Websites’ metadata refer to the second level of paratextual information such as website’s political bias, size, and popularity.

3 https://mediabiasfactcheck.com/conspiracy/

4 Note that the final number of conspiracy websites in LOCO is 58. This is because during the data cleaning process for some websites we did not obtain any webpages (e.g., stormfront.org, learntherisk.org). Other websites were excluded because they were either collections of tweets and videos or were CT search engines (e.g., qanon.pub, disclose.tv, and alternativenews.com).

5 E.g., telegraph.co.uk from the URL https://www.telegraph.co.uk/news/uknews/1577644/MMR-vaccine-doesnt-cause-autism-says-study.html.

6 Note that this number is different from the final *N* = 92 for mainstream websites. This is because after the cleaning section, the website urbandictionary.com was no longer present.

7 URL: https://www.google.co.uk/search?q=site%3Abbc.com+moon+landing&hl=en

8 The discrepancy with Table 2, which shows the minimum word count as 97 words in a document, is due to the fact that at this stage (document cleaning) we counted words as portions of text separated by spaces, while LOCO’s final word count was performed with TAACO (see “Text statistics” section).

9 The word “conspir*” was chosen to be able to retrieve all conspiracy-related words (conspiracies, conspiracist, conspiracy, conspiration, conspirator, conspiratorial, conspiratorially, conspiratress, conspire, conspirer, and conspiring) but not others (e.g., conspicuous). This was checked on both American and British Oxford English dictionaries.

10 From this count measure, a Boolean measure of “mentioning conspiracy” can easily be derived by simply stating “TRUE if mentions > 0.”

11 We thank the anonymous reviewers for this suggestion.

12 see: https://developers.facebook.com/tools/debug/)

13 https://mediabiasfactcheck.com/methodology/

14 https://www.poynter.org/ifcn/

15 https://www.similarweb.com/corp/ourdata/

16 E.g., https://www.google.co.uk/search?q=site%3Abbc.com

17 Note that the sum of pro-science, least-biased, and biased documents is 57,575 (and not 72,806, the total of mainstream documents). This is because not all websites are rated by MBFC, and therefore documents from these websites do not compare in plot B.

## References

[CR1] Allen, C., & Murdock, J. (2020). *LDA Topic Modeling: Contexts for the History & Philosophy of Science*. http://philsci-archive.pitt.edu/17261/

[CR2] Aston, G., & Burnard, L. (1998). The BNC Handbook: Exploring the British National Corpus with SARA. In: *English Language and Linguistics*. Edinburgh University Press.

[CR3] Aupers S (2012). ‘Trust no one’: Modernization, paranoia and conspiracy culture. European Journal of Communication.

[CR4] AVAAZ. (2020). *Facebook’s Algorithm: A Major Threat to Public Health*. https://secure.avaaz.org/campaign/en/facebook_threat_health/

[CR5] Baly, R., Karadzhov, G., Alexandrov, D., Glass, J., & Nakov, P. (2018). Predicting Factuality of Reporting and Bias of News Media Sources. *Proceedings of the 2018 Conference on Empirical Methods in Natural Language Processing*, 3528–3539. 10.18653/v1/D18-1389

[CR6] Bangerter A, Wagner-Egger P, Delouvée S, Butter M, Knight P (2020). The Spread of Conspiracy Theories. *Routledge Handbook of Conspiracy Theories*.

[CR7] Barkun M (2017). President Trump and the “Fringe”. Terrorism and Political Violence.

[CR8] Baroni M, Bernardini S, Ferraresi A, Zanchetta E (2009). The WaCky wide web: a collection of very large linguistically processed web-crawled corpora. Language Resources and Evaluation.

[CR9] Barron ATJ, Huang J, Spang RL, DeDeo S (2018). Individuals, institutions, and innovation in the debates of the French Revolution. Proceedings of the National Academy of Sciences.

[CR10] Bates D, Mächler M, Bolker B, Walker S (2015). Fitting linear mixed-effects models using lme4. Journal of Statistical Software.

[CR11] Bessi A (2016). Personality traits and echo chambers on facebook. Computers in Human Behavior.

[CR12] Bessi A, Scala A, Rossi L, Zhang Q, Quattrociocchi W (2014). The economy of attention in the age of (mis)information. Journal of Trust Management.

[CR13] Bessi A, Coletto M, Davidescu GA, Scala A, Caldarelli G, Quattrociocchi W (2015). Science vs Conspiracy: Collective Narratives in the Age of Misinformation. PLOS ONE.

[CR14] Bessi A, Zollo F, Del Vicario M, Scala A, Caldarelli G, Quattrociocchi W (2015). Trend of Narratives in the Age of Misinformation. PLOS ONE.

[CR15] Betsch C, Ulshöfer C, Renkewitz F, Betsch T (2011). The Influence of Narrative v. Statistical Information on Perceiving Vaccination Risks. Medical Decision Making.

[CR16] Biddlestone M, Green R, Douglas KM (2020). Cultural orientation, power, belief in conspiracy theories, and intentions to reduce the spread of COVID-19. British Journal of Social Psychology.

[CR17] Bird S, Klein E, Loper E (2009). *Natural language processing with Python: analyzing text with the natural language toolkit*.

[CR18] Blei DM, Ng AY, Jordan MI (2003). Latent Dirichlet allocation. Journal of Machine Learning Research.

[CR19] Bogart LM, Wagner G, Galvan FH, Banks D (2010). Conspiracy Beliefs About HIV Are Related to Antiretroviral Treatment Nonadherence Among African American Men With HIV. JAIDS Journal of Acquired Immune Deficiency Syndromes.

[CR20] Brugnoli E, Cinelli M, Quattrociocchi W, Scala A (2019). Recursive patterns in online echo chambers. Scientific Reports.

[CR21] Brysbaert M, New B (2009). Moving beyond Kučera and Francis: A critical evaluation of current word frequency norms and the introduction of a new and improved word frequency measure for American English. Behavior Research Methods.

[CR22] Brysbaert M, Warriner AB, Kuperman V (2014). Concreteness ratings for 40 thousand generally known English word lemmas. Behavior Research Methods.

[CR23] Butter M, Knight P, Butter M, Knight P (2020). *Routledge Handbook of Conspiracy Theories*.

[CR24] Castelo, S., Santos, A., Almeida, T., Pham, K., Freire, J., Elghafari, A., & Nakamura, E. (2019). A topic-agnostic approach for identifying fake news pages. *The Web Conference 2019 - Companion of the World Wide Web Conference, WWW 2019*. 10.1145/3308560.3316739

[CR25] Cinelli M, De Francisci Morales G, Galeazzi A, Quattrociocchi W, Starnini M (2021). The echo chamber effect on social media. Proceedings of the National Academy of Sciences.

[CR26] Clarke S (2007). Conspiracy Theories and the Internet: Controlled Demolition and Arrested Development. Episteme.

[CR27] Cortese MJ, Fugett A (2004). Imageability ratings for 3,000 monosyllabic words. Behavior Research Methods, Instruments, & Computers.

[CR28] Crossley SA, Kyle K, McNamara DS (2016). The tool for the automatic analysis of text cohesion (TAACO): Automatic assessment of local, global, and text cohesion. Behavior Research Methods.

[CR29] Crossley SA, Kyle K, Dascalu M (2019). The Tool for the Automatic Analysis of Cohesion 2.0: Integrating semantic similarity and text overlap. Behavior Research Methods.

[CR30] de Vries E, Schoonvelde M, Schumacher G (2018). No Longer Lost in Translation: Evidence that Google Translate Works for Comparative Bag-of-Words Text Applications. Political Analysis.

[CR31] Del Vicario M, Bessi A, Zollo F, Petroni F, Scala A, Caldarelli G, Stanley HE, Quattrociocchi W (2016). The spreading of misinformation online. Proceedings of the National Academy of Sciences.

[CR32] Del Vicario M, Vivaldo G, Bessi A, Zollo F, Scala A, Caldarelli G, Quattrociocchi W (2016). Echo Chambers: Emotional Contagion and Group Polarization on Facebook. Scientific Reports.

[CR33] Del Vicario M, Scala A, Caldarelli G, Stanley HE, Quattrociocchi W (2017). Modeling confirmation bias and polarization. Scientific Reports.

[CR34] Douglas KM, Sutton RM (2011). Does it take one to know one? Endorsement of conspiracy theories is influenced by personal willingness to conspire. British Journal of Social Psychology.

[CR35] Douglas KM, Sutton RM (2018). Why conspiracy theories matter: A social psychological analysis. European Review of Social Psychology.

[CR36] Douglas KM, Uscinski JE, Sutton RM, Cichocka A, Nefes T, Ang CS, Deravi F (2019). Understanding Conspiracy Theories. Political Psychology.

[CR37] Eicher V, Bangerter A, Sammut G, Andreouli E, Gaskell G, Valsiner J (2015). Social representations of infectious diseases. *The Cambridge Handbook of Social Representations*.

[CR38] Einstein KL, Glick DM (2015). Do I Think BLS Data are BS? The Consequences of Conspiracy Theories. Political Behavior.

[CR39] Faasse K, Chatman CJ, Martin LR (2016). A comparison of language use in pro- and anti-vaccination comments in response to a high profile Facebook post. Vaccine.

[CR40] Fast, E., Chen, B., & Bernstein, M. S. (2016). Empath. *Proceedings of the 2016 CHI Conference on Human Factors in Computing Systems*, 4647–4657. 10.1145/2858036.2858535

[CR41] Fong A, Roozenbeek J, Goldwert D, Rathje S, van der Linden S (2021). The language of conspiracy: A psychological analysis of speech used by conspiracy theorists and their followers on Twitter. Group Processes & Intergroup Relations.

[CR42] Franks B, Bangerter A, Bauer MW (2013). Conspiracy theories as quasi-religious mentality: an integrated account from cognitive science, social representations theory, and frame theory. Frontiers in Psychology.

[CR43] Franks, B., Bangerter, A., Bauer, M. W., Hall, M., & Noort, M. C. (2017). Beyond “Monologicality”? Exploring Conspiracist Worldviews. *Frontiers in Psychology*, *8*. 10.3389/fpsyg.2017.00861 10.3389/fpsyg.2017.00861 PMC5476781 28676768

[CR44] Fry E (2000). *1000 instant words: the most common words for teaching reading, writing and spelling*.

[CR45] Fu LY, Zook K, Spoehr-Labutta Z, Hu P, Joseph JG (2016). Search Engine Ranking, Quality, and Content of Web Pages That Are Critical Versus Noncritical of Human Papillomavirus Vaccine. Journal of Adolescent Health.

[CR46] Golec de Zavala A, Cichocka A (2012). Collective narcissism and anti-Semitism in Poland. Group Processes & Intergroup Relations.

[CR47] Grün B, Hornik K (2011). Topicmodels: An R package for fitting topic models. Journal of Statistical Software.

[CR48] Guerini, M., Giampiccolo, D., Moretti, G., Sprugnoli, R., & Strapparava, C. (2013). The New Release of CORPS: A Corpus of Political Speeches Annotated with Audience Reactions. In: *Lecture Notes in Computer Science (including subseries Lecture Notes in Artificial Intelligence and Lecture Notes in Bioinformatics)* (pp. 86–98). 10.1007/978-3-642-41545-6_8

[CR49] Heath C, Bell C, Sternberg E (2001). Emotional selection in memes: The case of urban legends. Journal of Personality and Social Psychology.

[CR50] Hills TT (2019). The Dark Side of Information Proliferation. Perspectives on Psychological Science.

[CR51] Imhoff R, Lamberty P, Klein O (2018). Using Power as a Negative Cue: How Conspiracy Mentality Affects Epistemic Trust in Sources of Historical Knowledge. Personality and Social Psychology Bulletin.

[CR52] Imhoff R, Dieterle L, Lamberty P (2021). Resolving the Puzzle of Conspiracy Worldview and Political Activism: Belief in Secret Plots Decreases Normative but Increases Nonnormative Political Engagement. Social Psychological and Personality Science.

[CR53] Jensen T (2013). *Democrats and Republicans differ on conspiracy theory beliefs*.

[CR54] Jolley D, Douglas KM (2014). The social consequences of conspiracism: Exposure to conspiracy theories decreases intentions to engage in politics and to reduce one’s carbon footprint. British Journal of Psychology.

[CR55] Jolley D, Douglas KM (2014). The Effects of Anti-Vaccine Conspiracy Theories on Vaccination Intentions. PLoS ONE.

[CR56] Jolley D, Paterson JL (2020). Pylons ablaze: Examining the role of 5G COVID-19 conspiracy beliefs and support for violence. British Journal of Social Psychology.

[CR57] Jolley D, Douglas KM, Leite AC, Schrader T (2019). Belief in conspiracy theories and intentions to engage in everyday crime. British Journal of Social Psychology.

[CR58] Klein, C., Clutton, P., & Polito, V. (2018). Topic Modeling Reveals Distinct Interests within an Online Conspiracy Forum. *Frontiers in Psychology*, *9*. 10.3389/fpsyg.2018.00189 10.3389/fpsyg.2018.00189 PMC5826393 29515501

[CR59] Klein C, Clutton P, Dunn AG (2019). Pathways to conspiracy: The social and linguistic precursors of involvement in Reddit’s conspiracy theory forum. PLOS ONE.

[CR60] Kuperman V, Stadthagen-Gonzalez H, Brysbaert M (2012). Age-of-acquisition ratings for 30,000 English words. Behavior Research Methods.

[CR61] Kuznetsova, A., Brockhoff, P. B., & Christensen, R. H. B. (2017). lmerTest Package: Tests in Linear Mixed Effects Models. *Journal of Statistical Software*, *82*(13). 10.18637/jss.v082.i13

[CR62] Kwon S, Cha M, Jung K (2017). Rumor Detection over Varying Time Windows. PLOS ONE.

[CR63] Kyle K, Crossley SA, Kim YJ (2015). Native language identification and writing proficiency. International Journal of Learner Corpus Research.

[CR64] Lansdall-Welfare T, Sudhahar S, Thompson J, Lewis J, Cristianini N (2017). Content analysis of 150 years of British periodicals. Proceedings of the National Academy of Sciences.

[CR65] Lantian A, Muller D, Nurra C, Klein O, Berjot S, Pantazi M (2018). Stigmatized beliefs: Conspiracy theories, anticipated negative evaluation of the self, and fear of social exclusion. European Journal of Social Psychology.

[CR66] Lazarus, J. V., Ratzan, S. C., Palayew, A., Gostin, L. O., Larson, H. J., Rabin, K., Kimball, S., & El-Mohandes, A. (2020). A global survey of potential acceptance of a COVID-19 vaccine. *Nature Medicine*. 10.1038/s41591-020-1124-9 10.1038/s41591-020-1124-9 PMC7573523 33082575

[CR67] Li Y, Engelthaler T, Siew CSQ, Hills TT (2019). The Macroscope: A tool for examining the historical structure of language. Behavior Research Methods.

[CR68] Li Y, Hills T, Hertwig R (2020). A brief history of risk. Cognition.

[CR69] Marchlewska, M., Cichocka, A., & Kossowska, M. (2018). Addicted to answers: Need for cognitive closure and the endorsement of conspiracy beliefs. *European Journal of Social Psychology*. 10.1002/ejsp.2308

[CR70] Meppelink CS, Smit EG, Fransen ML, Diviani N (2019). “I was Right about Vaccination”: Confirmation Bias and Health Literacy in Online Health Information Seeking. Journal of Health Communication.

[CR71] Michel J-B, Shen YK, Aiden AP, Veres A, Gray MK, Pickett JP, Hoiberg D, Clancy D, Norvig P, Orwant J, Pinker S, Nowak MA, Aiden EL (2011). Quantitative Analysis of Culture Using Millions of Digitized Books. Science.

[CR72] Mitra, T., Counts, S., & Pennebaker, J. W. (2016). Understanding anti-vaccination attitudes in social media. *Proceedings of the 10th International Conference on Web and Social Media, ICWSM 2016*.

[CR73] Nguyen, D., Liakata, M., DeDeo, S., Eisenstein, J., Mimno, D., Tromble, R., & Winters, J. (2020). How We Do Things With Words: Analyzing Text as Social and Cultural Data. *Frontiers in Artificial Intelligence*, *3*. 10.3389/frai.2020.00062 10.3389/frai.2020.00062 PMC7861331 33733179

[CR74] Nikolov D, Oliveira DFM, Flammini A, Menczer F (2015). Measuring online social bubbles. PeerJ Computer Science.

[CR75] Okuhara T, Ishikawa H, Okada M, Kato M, Kiuchi T (2017). Readability comparison of pro- and anti-HPV-vaccination online messages in Japan. Patient Education and Counseling.

[CR76] Oliver JE, Wood TJ (2014). Conspiracy Theories and the Paranoid Style(s) of Mass Opinion. American Journal of Political Science.

[CR77] Ooms, J. (2019). *curl: A Modern and Flexible Web Client for R*. https://cran.r-project.org/package=curl

[CR78] Oswald S (2016). Conspiracy and bias: argumentative features and persuasiveness of conspiracy theories. OSSA Conference Archive.

[CR79] Pauwels K, Demirci C, Yildirim G, Srinivasan S (2016). The impact of brand familiarity on online and offline media synergy. International Journal of Research in Marketing.

[CR80] Pennycook G, Rand DG (2019). Fighting misinformation on social media using crowdsourced judgments of news source quality. Proceedings of the National Academy of Sciences.

[CR81] Perez JC, Montagnier L (2020). Covid-19, Sars And Bats Coronaviruses Genomes Peculiar Homologous RNA Sequences. International Journal of Research -GRANTHAALAYAH.

[CR82] R Core Team. (2019). *R: A Language and Environment for Statistical Computing*. https://www.r-project.org/

[CR83] Raab MH, Auer N, Ortlieb SA, Carbon C-C (2013). The Sarrazin effect: the presence of absurd statements in conspiracy theories makes canonical information less plausible. Frontiers in Psychology.

[CR84] Raab, M. H., Ortlieb, S. A., Auer, N., Guthmann, K., & Carbon, C.-C. (2013b). Thirty shades of truth: conspiracy theories as stories of individuation, not of pathological delusion. *Frontiers in Psychology*, *4*. 10.3389/fpsyg.2013.00406 10.3389/fpsyg.2013.00406 PMC3705173 23847576

[CR85] Risius, M., Aydinguel, O., & Haug, M. (2019). Towards an understanding of conspiracy echo chambers on Facebook. *Proceedings of the 27th European Conference on Information Systems (ECIS)*. https://aisel.aisnet.org/ecis2019_rip/36

[CR86] Sak G, Diviani N, Allam A, Schulz PJ (2015). Comparing the quality of pro- and anti-vaccination online information: a content analysis of vaccination-related webpages. BMC Public Health.

[CR87] Salmon DA, Moulton LH, Omer SB, DeHart MP, Stokley S, Halsey NA (2005). Factors associated with refusal of childhood vaccines among parents of school-aged children: a case-control study. Archives of Pediatrics & Adolescent Medicine.

[CR88] Samory, M., & Mitra, T. (2018a). Conspiracies online: User discussions in a conspiracy community following dramatic events. *12th International AAAI Conference on Web and Social Media, ICWSM 2018*.

[CR89] Samory, M., & Mitra, T. (2018b). “The Government Spies Using Our Webcams:” The language of conspiracy theories in online discussions. *Proceedings of the ACM on Human-Computer Interaction*, *2*(152). 10.1145/3274421

[CR90] Smith N, Graham T (2019). Mapping the anti-vaccination movement on Facebook. Information, Communication & Society.

[CR91] Sternisko A, Cichocka A, Van Bavel JJ (2020). The dark side of social movements: social identity, non-conformity, and the lure of conspiracy theories. Current Opinion in Psychology.

[CR92] Stroud NJ (2010). Polarization and Partisan Selective Exposure. Journal of Communication.

[CR93] Swami V, Barron D, Weis L, Furnham A (2018). To Brexit or not to Brexit: The roles of Islamophobia, conspiracist beliefs, and integrated threat in voting intentions for the United Kingdom European Union membership referendum. British Journal of Psychology.

[CR94] Tausczik YR, Pennebaker JW (2010). The psychological meaning of words: LIWC and computerized text analysis methods. Journal of Language and Social Psychology.

[CR95] Uscinski, J. E., Parent, J. M., & Torres, B. (2011). Conspiracy Theories Are for Losers. *APSA 2011 Annual Meeting Paper*. https://ssrn.com/abstract=1901755

[CR96] Uscinski, J. E., DeWitt, D., & Atkinson, M. D. (2018). A Web of Conspiracy? Internet and Conspiracy Theory. In: *Handbook of Conspiracy Theory and Contemporary Religion* (pp. 106–130). BRILL. 10.1163/9789004382022_007

[CR97] van der Linden S (2015). The conspiracy-effect: Exposure to conspiracy theories (about global warming) decreases pro-social behavior and science acceptance. Personality and Individual Differences.

[CR98] van Prooijen J-W, Krouwel APM, Pollet TV (2015). Political Extremism Predicts Belief in Conspiracy Theories. Social Psychological and Personality Science.

[CR99] von Luxburg U, Williamson RC, Guyon I, Guyon I, Dror G, Lemaire V, Taylor G, Silver D (2012). Clustering: Science or Art?. *Proceedings of ICML Workshop on Unsupervised and Transfer Learning*.

[CR100] Vosoughi S, Roy D, Aral S (2018). The spread of true and false news online. Science.

[CR101] Wakefield A, Murch S, Anthony A, Linnell J, Casson D, Malik M, Berelowitz M, Dhillon A, Thomson M, Harvey P, Valentine A, Davies S, Walker-Smith J (1998). RETRACTED: Ileal-lymphoid-nodular hyperplasia, non-specific colitis, and pervasive developmental disorder in children. The Lancet.

[CR102] Warriner AB, Kuperman V, Brysbaert M (2013). Norms of valence, arousal, and dominance for 13,915 English lemmas. Behavior Research Methods.

[CR103] Westen D, Blagov PS, Harenski K, Kilts C, Hamann S (2006). Neural Bases of Motivated Reasoning: An fMRI Study of Emotional Constraints on Partisan Political Judgment in the 2004 U.S. Presidential Election. Journal of Cognitive Neuroscience.

[CR104] Wood MJ (2018). Propagating and Debunking Conspiracy Theories on Twitter During the 2015–2016 Zika Virus Outbreak. Cyberpsychology, Behavior, and Social Networking.

[CR105] Wood MJ, Douglas KM (2013). What about building 7? A social psychological study of online discussion of 9/11 conspiracy theories. Frontiers in Psychology.

[CR106] Wood, M. J., & Douglas, K. M. (2015). Online communication as a window to conspiracist worldviews. *Frontiers in Psychology*, *6*. 10.3389/fpsyg.2015.00836 10.3389/fpsyg.2015.00836 PMC4470066 26136717

[CR107] Zannettou, S., Bradlyn, B., De Cristofaro, E., Kwak, H., Sirivianos, M., Stringini, G., & Blackburn, J. (2018). What is Gab. *Companion of the Web Conference 2018 on The Web Conference 2018 - WWW ’18*, 1007–1014. 10.1145/3184558.3191531

[CR108] Zollo, F., Bessi, A., Del Vicario, M., Scala, A., Caldarelli, G., Shekhtman, L., Havlin, S., & Quattrociocchi, W. (2017). Debunking in a world of tribes. *PLoS ONE*. 10.1371/journal.pone.0181821 10.1371/journal.pone.0181821 PMC5524392 28742163

[CR109] Zubiaga A, Liakata M, Procter R, Wong Sak Hoi G, Tolmie P (2016). Analysing How People Orient to and Spread Rumours in Social Media by Looking at Conversational Threads. PLOS ONE.

