# Supplementary material for: LOCO: The 88-million-word language of conspiracy corpus
Source: Behav Res Methods. 2021 Oct 25;54(4):1794–817. doi: 10.3758/s13428-021-01698-z (PMC8545361; doi:10.3758/s13428-021-01698-z)
Supplement: Supplementary file 1 — (PDF 7022 kb) [file 13428_2021_1698_MOESM1_ESM.pdf]

LOCO: the 88-million-word language of conspiracy corpus

SUPPLEMENTAL MATERIAL

Alessandro Miani[1], Thomas Hills[2,3], and Adrian Bangerter[1]

[1] *Institute of Work and Organizational Psychology, University of Neuchâtel, Rue Emile-Argand 11, 2000 Neuchâtel, Switzerland*
[2] *Department of Psychology, University of Warwick, University Road, Coventry CV47AL, United Kingdom*
[3] *The Alan Turing Institute, British Library, 96 Euston Road, London, NW1 2DB, United Kingdom*

Contents

1 Evaluation of conspiracy and mainstream labels **3**

2 URL extraction workflow **4**

3 Testing boilerplate stripping **5**

4 LIWC and Empath correlations **6**

5 Text preprocessing **7**
5.1 Identifying stopwords . . . 7
5.2 Cleaning pipeline . . . 7

6 Topic description **10**
6.1 Top term beta values . . . 12

7 Comparison across 3 ks **13**
7.1 Topic specificity across different ks . . . 20

8 Representativeness of conspiracy documents **21**

9 Popularity and spread metrics correlations **24**

10 Lexical features of mentioning conspiracy **26**

11 Properties of representative conspiracy documents **29**

SM 1 Evaluation of conspiracy and mainstream labels

In order to estimate to what extent conspiracy and mainstream documents reflect their true labels, namely whether a document is in fact either conspiracy or mainstream, we have visually inspected and classified a subset of documents. We extracted a random sample of 60 conspiracy and 60 mainstream documents. One of the authors (AM) manually coded these documents either as conspiracy or mainstream. Such coding was performed blind, meaning that only unformatted text with no other information was available to the coder (besides a newly generated random ID to allow documents to match with true labels after the manual coding). Results show that ratings had an overall accuracy of .88 (95%CIs = .81 - .93, Cohen's k = .77), with a sensitivity (true positive rate: conspiracy correctly labelled as conspiracy; 9 misclassified) of .85 and specificity (true negative rate: mainstream correctly labelled as mainstream; 5 misclassified) of .92.

SM 2 URL extraction workflow

The URL extraction was performed via the following workflow (see Figure S1):

1. Aggregate seeds and websites into a unique query
2. Send query to Google and extract links from the first page
3. If error, wait (to avoid the "HTTP 429 too many requests" error) and repeat (no more than five times)
4. If more than 10 results are returned, go to the next page; if error, wait
5. If less than 10 results are returned, store results and move to the next query

Figure S1 – URL extraction workflow

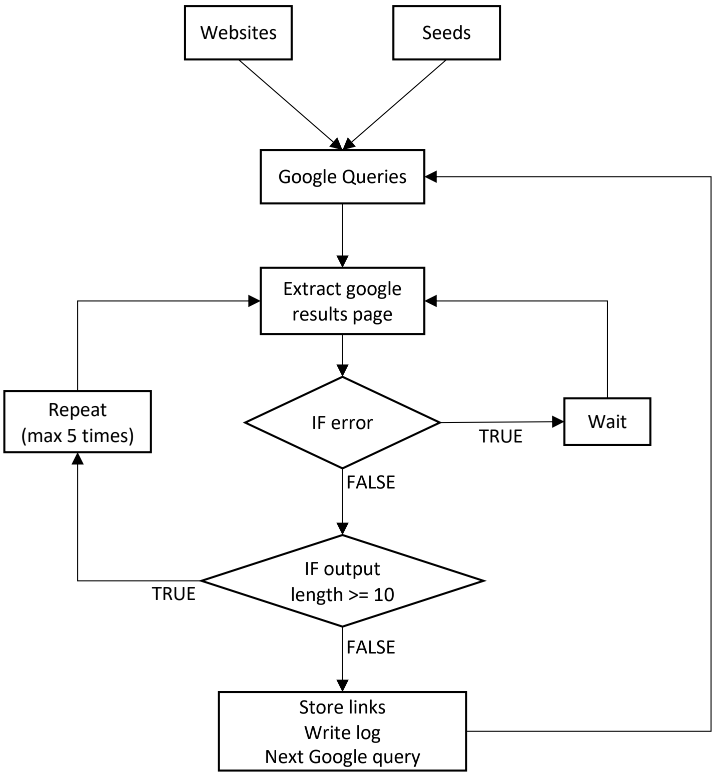

SM 3 Testing boilerplate stripping

In order to find the most reliable script (*i.e.*, the one that returns a cleaned text most similar to manual cleaning), we tested five different Python packages: *beautifulsoup*, *boilerpipe*, *Goose*, *HTML2text*, and *justext*. To test these scripts, we visually inspected a set of 100 random links and, when possible, we manually extracted the useful portions of text (*i.e.*, copying the text and pasting it to a new file). This was done on 64 webpages from 39 unique websites. Then, we ran each of the five scripts along the URL list. Doing so, we obtained six versions of the text per each webpage (one from manual extraction, and five from the scripts). We then tested how each script was similar to the manually extracted text on different metrics. These were: a) the correlation of number of characters and number of words, b) Jaccard similarity (*i.e.*, 1-distance) on different levels of ngrams (1, 5, 10, 50, 100), and c) the percentage of non-empty texts returned by the script. Per each set of metrics, we extracted values on the individual text-level and then we averaged them into a single value per metric per boilerplate. The Goose package (`https://github.com/grangier/python-goose`) returned the best performance (highest aggregated mean of all metrics, see Figure S2) and therefore was chosen for extracting the texts.

Figure S2 – Boilerplate stripping test results

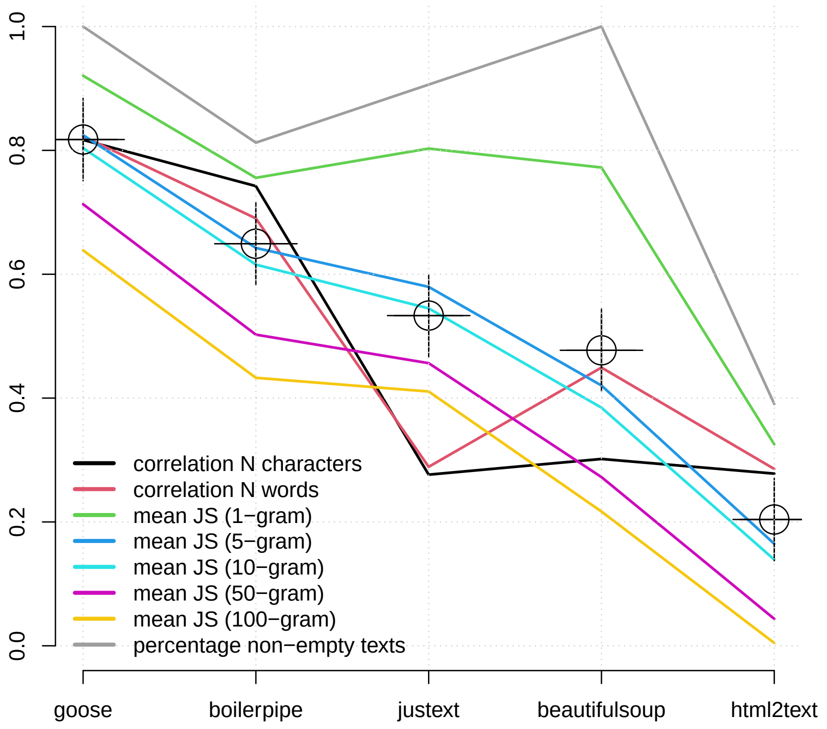

Note. Comparisons for each of the 5 stripping packages we tested on the 8 different metrics described in the text. The circled crosses represent the mean of the 8 metrics within stripping package. JS: Jaccard similarity.

SM 4 LIWC and Empath correlations

Here we report the top 20 (positive and negative) highest and lowest absolute correlations between LIWC and Empath dictionaries. Correlations have been run at the document level (N = 96,743). See Table S1 below.

Table S1 – LIWC and Empath correlations

	highest			*lowest (abs)*			*lowest*	
**LIWC**	**Empath**	**r**	**LIWC**	**Empath**	**r**	**LIWC**	**Empath**	**r**
money	valuable	0.98	article	disappointment	0	Tone	violence	-0.58
money	payment	0.98	Colon	pet	0	body	leader	-0.58
health	health	0.98	discrep	aggression	0	tentat	leader	-0.58
money	banking	0.98	drives	lust	0	Tone	weapon	-0.59
money	money	0.97	netspeak	government	0	percept	gain	-0.59
family	family	0.97	Comma	exercise	0	body	government	-0.59
money	economics	0.97	swear	terrorism	0	Tone	suffering	-0.60
health	medical_emergency	0.97	space	fight	0	percept	government	-0.60
relig	worship	0.97	reward	noise	0	cause	party	-0.60
family	children	0.95	Colon	cooking	0	shehe	science	-0.61
money	wealthy	0.94	Dash	fire	0	number	speaking	-0.62
body	body	0.94	shehe	ugliness	0	male	science	-0.62
relig	religion	0.93	number	leisure	0	cause	home	-0.62
relig	divine	0.93	SemiC	banking	0	Analytic	confusion	-0.62
money	negotiate	0.92	health	restaurant	0	Sixltr	friends	-0.62
death	kill	0.91	social	hipster	0	cause	wedding	-0.63
work	business	0.91	female	anonymity	0	Sixltr	childish	-0.63
home	domestic_work	0.91	they	dispute	0	Analytic	speaking	-0.66
bio	health	0.90	nonflu	weather	0	Tone	aggression	-0.67
leisure	fun	0.90	ipron	death	0	Tone	kill	-0.68

SM 5 Text preprocessing

Text preprocessing for topic extraction relied on the corpus' document-term matrix (DTM), namely a bag of words for the whole corpus that stores the occurrences of each word (columns) for each document (rows). Before generating the DTM, it was needed to pre-process each text so to remove noise, reduce sparsity, while reducing computation time. This preprocessing was mostly done by removing the most frequent (*e.g.*, stopwords) and infrequent (*e.g.*, misspellings or extreme rare) words.

5.1 Identifying stopwords

Before running the cleaning pipeline, we first identified a set of stopwords suitable for our purpose. We started by merging the 175 English stopwords from the *stopwords* R package (Benoit et al., 2020) with the top 100 most frequent words in English (Fry, 2000) and single letters (N = 26). This resulted in a set of 229 words (see Table S2 below). Motivated from the literature, from this set, we decide to remove (*e.g.*, include in DTM) the following terms:

1. Pronouns: in line with research on social motives underlying belief in CTs (Douglas et al., 2019), the conspiratorial language heavily relies on pronouns such as we/they marking a process of social identification (we) by exclusion from the outgroup (they). This list (of pronouns) was compiled from the LIWC category pronouns.

2. Negations and interrogative words: crucial for the rhetoric of conspiracy (Oswald, 2016) based on refutation and questioning. Negations were extracted from the LIWC category negations, while wh-question words were manually excluded by looking at the remaining list of stopwords.

3. Ad hoc set of words (within the stopwords) that might be important for conspiracy language. This was done by visually inspecting the list of stopwords and removing those potentially important such as against (language of fighting), *oil* (related to economy), *people* (see *we/they* above) as well as *see* and *write* (reporting facts).

Note that we also removed stopwords that included contractions (*e.g.*, *I'm*, *you're*) because in in the text preprocessing we expanded these contractions. The reasoning to expand contractions is that by keeping them, the term *they're* is treated as a different term than *they.* Because we were interested in keeping pronouns, expanding contractions allows us to keep them in the DTM. Furthermore, we have no theoretical reason to prefer a contracted vs an expanded form. Contractions were expanded by using the dataset contractions (N = 70 entries) from the R package *qdapDictionaries* (Rinker, 2013).

5.2 Cleaning pipeline

Once the list of stopwords was compiled, we proceeded with the text cleaning pipeline. This was done by using the *quanteda* R package (Benoit et al., 2018). The pipeline was as follow:

1. Remove non-ASCII characters

2. Tolower (*i.e.*, convert upper case to lower case)

3. Tokenization
    (a) remove URLs
    (b) remove punctuation
    (c) remove numbers
    (d) remove separators
    (e) split hyphens
    (f) remove symbols
4. Expand contractions
5. Remove stopwords (see section SM 5.1)
6. Wordstem (words were reduced to their root, *e.g.*, "frequenc" for "frequency" and "frequencies")
7. Generate DTM
8. Trim the DTM to top 10,000 terms

The DTM was trimmed to the top 10,000 terms so to reduce sparsity and computation time. This reduced sparsity from 0.999 to 0.974. The DTM was finally composed of 96,743 documents and 10,000 terms, for a total of 47,523,008 types (without trimming, the DTM was 349,063 terms accounting for 50,547,244 types).

Table S2 – Stopword sets

Initial set (N = 229)	a, about, above, after, again, against, all, am, an, and, any, are, aren't, as, at, b, be, because, been, before, being, below, between, both, but, by, c, call, can, can't, cannot, come, could, couldn't, d, day, did, didn't, do, does, doesn't, doing, don't, down, during, e, each, f, few, find, first, for, from, further, g, get, go, h, had, hadn't, has, hasn't, have, haven't, having, he, he'd, he'll, he's, her, here, here's, hers, herself, him, himself, his, how, how's, i, i'd, i'll, i'm, i've, if, in, into, is, isn't, it, it's, its, itself, j, k, l, let's, like, long, look, m, made, make, many, may, me, more, most, mustn't, my, myself, n, no, nor, not, now, number, o, of, off, oil, on, once, one, only, or, other, ought, our, ours, ourselves, out, over, own, p, part, people, q, r, s, said, same, see, shan't, she, she'd, she'll, she's, should, shouldn't, so, some, such, t, than, that, that's, the, their, theirs, them, themselves, then, there, there's, these, they, they'd, they'll, they're, they've, this, those, through, time, to, too, two, u, under, until, up, use, v, very, w, was, wasn't, water, way, we, we'd, we'll, we're, we've, were, weren't, what, what's, when, when's, where, where's, which, while, who, who's, whom, why, why's, will, with, won't, word, would, wouldn't, write, x, y, you, you'd, you'll, you're, you've, your, yours, yourself, yourselves, z
To remove (N = 53)	**Pronouns**: i, me, my, myself, we, let, us, our, ours, ourselves, you, thou, thoust, thee, thy, thyself, your, yours, yourself, yourselves, she, he, her, hers, his, him, himself, hisself, herself, their, theirs, them, themself, themselves, they. **Negations and wh-question words**: not, cannot, no, nor, nope, none, what, when, where, which, who, how, why. **Ad hoc words**: against, oil, people, see, write
Final set (N = 137)	a, about, above, after, again, all, am, an, and, any, are, as, at, b, be, because, been, before, being, below, between, both, but, by, c, call, can, come, could, d, day, did, do, does, doing, down, during, e, each, f, few, find, first, for, from, further, g, get, go, h, had, has, have, having, here, if, in, into, is, it, its, itself, j, k, l, like, long, look, m, made, make, many, may, more, most, n, now, number, o, of, off, on, once, one, only, or, other, ought, out, over, own, p, part, q, r, s, said, same, should, so, some, such, t, than, that, the, then, there, these, this, those, through, time, to, too, two, u, under, until, up, use, v, very, w, was, water, way, were, while, whom, will, with, word, would, x, y, z

SM 6 Topic description

We extracted three sets of topic distributions based on different k (*i.e.*, number of topics) resolutions, namely $k = 100$, $k = 200$, and $k = 300$. As a consequence, LOCO is provided with a total of 600 topics. Besides the actual matrix with the gamma vales for each topic per each document (`topic_gamma.json`), we also attach to LOCO a dataset (with 600 entries, namely the number of topics) containing a description of each topic. Below, in Table S3, an excerpt of the file `topic_gamma.json`. Descriptions include:

1. **Topic ID**: the topic indexed as the sequential topic ID preceded by the k value, so for example "k100_4" refers to the fourth topic extracted with $k = 100$
2. **Top 15 words**: the top 15 words of the topic ordered by decreasing beta weight (see Figure S3)
3. **N (C/M)**: The total (N) number of documents whose such topic has the max gamma value across all topics within a k set. N_C and N_M refer to the number of documents within conspiracy and mainstream subcorpora. The variable "Prop conspiracy" refers to the proportion of conspiracy documents.
4. **Topic in K r**: It reports the topic with the highest correlation among all topics within the k set. The correlation is computed on the document level (N = 96,743).
5. **Topic all r**: same as above, but correlation is computed for all topics for all k sets (*i.e.*, N = 600) on the document level (N = 96,743).
6. **LF r**: the highest correlation with lexical features (LF) from Empath (E) and LIWC (L).

Table S3 – Excerpt from data frame `topic_description.json`

Topic ID	Top 15 words	N Total	N C	N M	Prop conspiracy	Topic In K	Topic In K r	Topic all	Topic all r	LF	LF r
k100_1	*studi, risk, increas, report, associ, case, group, age, data, effect, rate, result, factor, not, includ*	672	53	619	0.079	k100_42	0.15	k200_1	0.82	E_health	0.24
k100_2	*game, team, play, player, sport, world, year, win, event, their, club, who, season, match, footbal*	842	27	815	0.032	k100_89	0.12	k200_179	0.93	E_sports	0.42
k200_1	*studi, data, age, associ, rate, report, case, estim, among, popul, use, compar, differ, group, incid*	436	26	410	0.060	k200_196	0.14	k100_1	0.82	L_Parenth	0.22
k200_2	*gate, bill, foundat, world, microsoft, melinda, his, billion, billionair, founder, global, million, wealth, who, year*	766	157	609	0.205	k200_170	0.11	k300_294	0.85	L_home	0.32
k300_1	*inform, provid, requir, applic, avail, includ, not, servic, section, must, follow, assess, addit, date, request*	289	18	271	0.062	k300_300	0.11	k200_44	0.82	E_office	0.22
k300_2	*fluorid, teeth, dental, drink, tooth, health, decay, toothpast, dentist, children, suppli, brush, caviti, level, effect*	819	188	631	0.230	k300_122	0.14	k200_13	0.98	E_hygiene	0.57

6.1 Top term beta values

Note that we do not provide labels for topics. Instead, as it can be observed from the plots in Figure S3 below (and as suggested in Nguyen et al. 2020), it is possible to infer the topic content by inspecting the top 5 terms in each topic, which account for the majority of beta weights. In other words, each topic is mostly characterized by the top 5 terms while the contribution of the other terms to the topic is minimal.

Figure S3 – Distribution of beta weights across ks

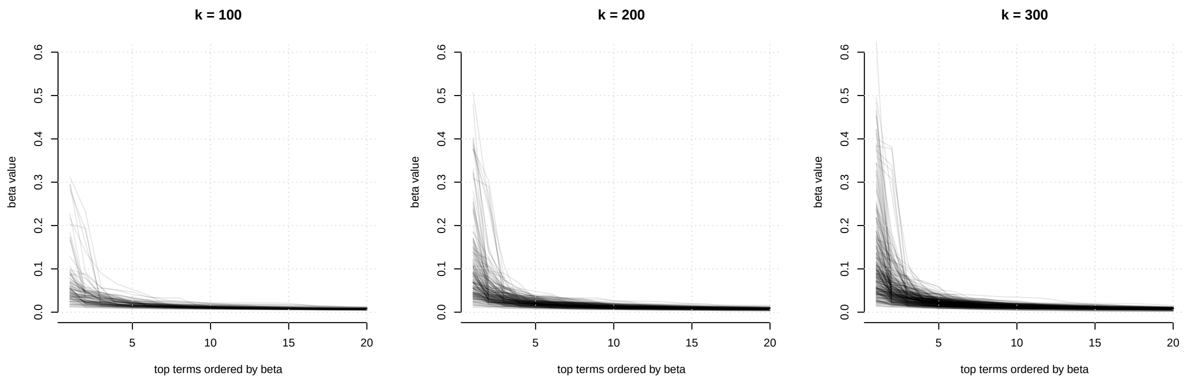

Note. Each line represents the ordered (decreasing from left to right) beta values for the top 20 terms for one of the k topics (from 100, left, to 300, right).

SM 7 Comparison across 3 ks

As mentioned in the main text (see section 3.6), we extracted three sets of topic distributions based on different k (*i.e.*, number of topics) resolutions, namely $k = 100$, $k = 200$, and $k = 300$. For illustrative purposes (not exhaustive), here, we selected a small set of themes to explore the extent to which similar topics (*i.e.*, topics revolving around the same theme, *e.g.*, Lady Diana's death) correlate, at the document level, on different levels of k resolution. For each theme, we selected a keyword capable of retrieving the associated topic in each k dataset by searching the keyword within the top 15 topic terms. For example, the keyword "mh370" retrieves the topics associated with the Malaysia Airlines Flight 370 that disappeared on 8 March 2014.

The set of keywords (associated with topic) we used was: *diana* (Lady Diana), *sandi* (Sandy Hook school shooting), *tower* (9/11 terroristic attack), *ebola* (epidemic), *mh370* (flight disappeared), *laden* (Osama Bin Laden), *jackson* (Michael Jackson), corona (covid-19 epidemic), *zika* (epidemic), *saddam* (Saddam Hussein), *elvi* (Elvis Presley), *5g* (5G communication technology).

In the following, for each theme, we present a descriptive table followed by a correlation matrix. In the descriptive table, each line describes the topics that revolve around the theme (see description of columns in section SM 6). For example, in Table S4 that contains topics revolving around Lady Diana, the two topics in k200 correlate with each other at r=.17, these are the highest correlations for the topics 107 and 189 within the k200 dataset. Similarly, within the k200 dataset, topics 107 and 189 correlate the most with the categories of *car* and *royalty* (from the Empath dictionary), respectively, suggesting that while topic 107 refers to Lady Diana's death (car accident), the topic 189 refers to the British Royal Family, possibly her life.

Table S4 – Topic description: Lady Diana

**K_Topic**	**Top 15 words**	**N C**	**N M**	**Max Topic**	**r**	**Max LF**	**r**
k100_88	*diana, princ, royal, princess, her, queen, harri, british, william, charl, death, famili, their, london, his*	168	1014	Topic_027	0.17	E_royalty	0.65
k200_107	*car, paul, crash, diana, driver, drive, accid, vehicl, fay, death, polic, pari, who, they, french*	99	293	Topic_189	0.17	E_car	0.36
k200_189	*diana, princ, royal, her, queen, harri, princess, charl, william, british, famili, elizabeth, palac, death, she*	79	727	Topic_107	0.17	E_royalty	0.7
k300_65	*diana, princ, princess, her, harri, royal, charl, death, william, fay, crash, she, british, palac, car*	125	827	Topic_258	0.16	E_royalty	0.55

Table S5 – Topic description: Lady Diana (correlation matrix)

	k100_88	k200_107	k200_189	k300_65
k100_88	1			
k200_107	0.56	1		
k200_189	0.88	0.17	1	
k300_65	0.93	0.63	0.78	1

Table S6 – Topic description: Sandy Hook

**K_Topic**	**Top 15 words**	**N C**	**N M**	**Max Topic**	**r**	**Max LF**	**r**
k100_97	*gun, shoot, polic, school, kill, hook, sandi, violenc, mass, who, shot, victim, year, children, peopl*	282	1143	Topic_043	0.12	E_weapon	0.6
k200_141	*gun, shoot, school, hook, sandi, kill, mass, violenc, lanza, shot, polic, connecticut, newtown, massacr, victim*	243	1037	Topic_096	0.14	E_weapon	0.57
k300_290	*gun, shoot, school, hook, sandi, kill, mass, violenc, lanza, connecticut, newtown, massacr, elementari, victim, firearm*	187	946	Topic_090	0.15	E_weapon	0.55

Table S7 – Topic description: Sandy Hook (correlation matrix)

	k100_97	k200_141	k300_290
k100_97	1		
k200_141	0.96	1	
k300_290	0.94	0.97	1

Table S8 – Topic description: 9/11 terroristic attack

K_Topic	Top 15 words	N C	N M	Max Topic	r	Max LF	r
k100_79	*attack, new, world, center, tower, build, york, septemb, trade, fire, who, event, peopl, terrorist, explos*	267	691	Topic_061	0.09	E_terrorism	0.22
k200_6	*attack, tower, center, septemb, trade, build, world, hijack, terrorist, york, new, pentagon, collaps, sept, plane*	276	516	Topic_169	0.2	E_terrorism	0.21
k300_72	*attack, tower, center, trade, world, septemb, new, york, pentagon, build, wtc, who, twin, ground, plane*	127	517	Topic_236	0.23	E_terrorism	0.16

Table S9 – Topic description: 9/11 terroristic attack (correlation matrix)

	k100_79	k200_6	k300_72
k100_79	1		
k200_6	0.92	1	
k300_72	0.89	0.92	1

Table S10 – Topic description: Ebola

K_Topic	Top 15 words	N C	N M	Max Topic	r	Max LF	r
k100_37	*ebola, outbreak, health, who, case, diseas, peopl, west, virus, spread, africa, epidem, countri, emerg, congo*	211	1570	Topic_091	0.1	E_medical_emergency	0.23
k200_159	*ebola, outbreak, health, who, case, diseas, virus, peopl, west, spread, congo, epidem, africa, worker, infect*	197	1471	Topic_115	0.12	E_medical_emergency	0.21
k300_62	*ebola, outbreak, health, virus, diseas, congo, peopl, case, west, spread, who, africa, epidem, worker, liberia*	174	1412	Topic_078	0.24	E_medical_emergency	0.2

Table S11 – Topic description: Ebola (correlation matrix)

	k100_37	k200_159	k300_62
k100_37	1		
k200_159	0.97	1	
k300_62	0.96	0.98	1

Table S12 – Topic description: MH370

K_Topic	Top 15 words	N C	N M	Max Topic	r	Max LF	r
k100_51	*plane, flight, search, airlin, aircraft, air, pilot, passeng, malaysia, crash, ocean, miss, fli, found, mh370*	144	1074	Topic_091	0.07	E_air_travel	0.63
k200_142	*search, plane, malaysia, flight, ocean, mh370, miss, disappear, airlin, found, malaysian, investig, debri, indian, aircraft*	55	886	Topic_123	0.19	E_air_travel	0.5
k300_272	*search, plane, malaysia, mh370, ocean, flight, miss, malaysian, disappear, found, airlin, investig, indian, debri, area*	48	847	Topic_187	0.21	E_air_travel	0.49

Table S13 – Topic description: MH370 (correlation matrix)

	k100_51	k200_142	k300_272
k100_51	1		
k200_142	0.94	1	
k300_272	0.93	0.99	1

Table S14 – Topic description: Osama Bin Laden

K_Topic	Top 15 words	N C	N M	Max Topic	r	Max LF	r
k100_61	*bin, laden, al, attack, terror, terrorist, saudi, qaeda, afghanistan, osama, pakistan, kill, islam, muslim, u.*	390	1378	Topic_045	0.11	L_anger	0.27
k200_34	*bin, laden, al, osama, qaeda, pakistan, his, kill, he, leader, attack, pakistani, offici, afghanistan, raid*	179	986	Topic_140	0.27	L_anger	0.19
k300_50	*bin, laden, al, osama, qaeda, afghanistan, pakistan, kill, taliban, attack, leader, his, he, pakistani, raid*	193	1048	Topic_227	0.17	E_war	0.2

Table S15 – Topic description: Osama Bin Laden (correlation matrix)

	k100_61	k200_34	k300_50
k100_61	1		
k200_34	0.89	1	
k300_50	0.91	0.97	1

Table S16 – Topic description: Michael Jackson

K_Topic	Top 15 words	N C	N M	Max Topic	r	Max LF	r
k100_15	*jackson, his, michael, he, death, murray, not, him, angel, singer, who, los, pop, die, estat*	43	912	Topic_043	0.1	L_male	0.22
k200_45	*jackson, michael, his, he, murray, death, singer, him, pop, angel, propofol, who, los, not, estat*	26	862	Topic_096	0.09	L_male	0.2
k300_18	*jackson, murray, he, his, death, michael, singer, doctor, not, propofol, him, los, dr, angel, aeg*	4	429	Topic_231	0.12	E_occupation	0.2
k300_231	*jackson, michael, his, he, death, pop, estat, music, him, who, fan, king, singer, star, alleg*	23	502	Topic_018	0.12	L_male	0.18

Table S17 – Topic description: Michael Jackson (correlation matrix)

	k100_15	k200_45	k300_18	k300_231
k100_15	1			
k200_45	0.98	1		
k300_18	0.81	0.84	1	
k300_231	0.63	0.61	0.12	1

Table S18 – Topic description: Coronavirus

K_Topic	Top 15 words	N C	N M	Max Topic	r	Max LF	r
k100_67	*vaccin, trial, develop, covid, test, clinic, phase, dose, coronavirus, research, against, work, effect, candid, treatment*	176	1938	Topic_072	0.14	E_health	0.27
k100_70	*coronavirus, covid, case, pandem, test, health, peopl, death, new, countri, virus, lockdown, confirm, week, report*	524	1864	Topic_099	0.2	L_number	0.16
k100_72	*virus, infect, diseas, test, flu, coronavirus, sar, spread, human, caus, immun, influenza, peopl, infecti, cov*	374	1047	Topic_035	0.19	E_health	0.29
k200_24	*coronavirus, covid, pandem, virus, health, peopl, outbreak, spread, lockdown, diseas, who, wuhan, public, social, infect*	260	441	Topic_028	0.34	E_health	0.15
k200_144	*vaccin, develop, trial, test, covid, dose, phase, candid, coronavirus, work, research, against, clinic, compani, immun*	126	1456	Topic_139	0.11	E_health	0.23
k300_200	*coronavirus, covid, pandem, virus, sar, cov, outbreak, spread, wuhan, peopl, infect, health, novel, diseas, respiratori*	113	230	Topic_119	0.22	E_health	0.18
k300_288	*coronavirus, covid, pandem, peopl, lockdown, virus, infect, reopen, health, week, countri, case, distanc, test, outbreak*	293	1225	Topic_110	0.3	L_relativ	0.15
k300_289	*vaccin, develop, trial, phase, test, dose, candid, covid, compani, clinic, coronavirus, work, human, moderna, research*	106	1254	Topic_036	0.17	E_health	0.2

Table S19 – Topic description: Coronavirus (correlation matrix)

	k100_67	k100_70	k100_72	k200_24	k200_144	k300_200	k300_288	k300_289
k100_67	1							
k100_70	0.03	1						
k100_72	0.14	0.14	1					
k200_24	0.14	0.73	0.31	1				
k200_144	0.92	0.02	0.12	0.08	1			
k300_200	0.22	0.4	0.55	0.71	0.15	1		
k300_288	0.02	0.79	0.05	0.51	0.01	0.07	1	
k300_289	0.88	0.02	0.09	0.08	0.96	0.12	0.01	1

Table S20 – Topic description: Zika Virus

**K_Topic**	**Top 15 words**	**N C**	**N M**	**Max Topic**	**r**	**Max LF**	**r**
k100_7	*zika, virus, mosquito, infect, case, brazil, diseas, health, travel, spread, pregnant, born, women, microcephali, area*	138	1363	Topic_072	0.04	E_programming	0.27
k200_14	*zika, virus, mosquito, infect, case, spread, travel, health, microcephali, pregnant, diseas, dengu, brazil, born, transmiss*	123	1277	Topic_199	0.06	E_programming	0.27
k300_169	*zika, virus, mosquito, infect, spread, microcephali, case, dengu, brazil, pregnant, travel, born, diseas, area, outbreak*	119	1200	Topic_078	0.12	E_programming	0.26

Table S21 – Topic description: Zika Virus (correlation matrix)

	k100_7	k200_14	k300_169
k100_7	1		
k200_14	0.98	1	
k300_169	0.96	0.98	1

Table S22 – Topic description: Saddam Hussein

**K_Topic**	**Top 15 words**	**N C**	**N M**	**Max Topic**	**r**	**Max LF**	**r**
k100_45	*iraq, iran, saddam, israel, iraqi, war, hussein, syria, state, isra, against, u., al, arab, regim*	695	1209	Topic_071	0.21	E_war	0.38
k200_89	*iraq, saddam, iraqi, hussein, al, his, baghdad, he, war, regim, forc, arab, invas, kuwait, against*	93	916	Topic_094	0.17	E_war	0.25
k300_134	*iraq, saddam, iraqi, hussein, al, his, baghdad, he, regim, war, arab, invas, forc, dictat, leader*	54	865	Topic_102	0.15	E_war	0.24

Table S23 – Topic description: Saddam Hussein (correlation matrix)

	k100_45	k200_89	k300_134
k100_45	1		
k200_89	0.76	1	
k300_134	0.74	0.98	1

Table S24 – Topic description: Elvis Presley

**K_Topic**	**Top 15 words**	**N C**	**N M**	**Max Topic**	**r**	**Max LF**	**r**
k100_78	*gate, elvi, his, bill, presley, he, year, king, microsoft, world, who, melinda, fan, vega, memphi*	81	1144	Topic_066	0.18	L_home	0.25
k200_46	*elvi, presley, his, he, king, fan, vega, memphi, las, graceland, year, rock, record, roll, music*	6	1079	Topic_131	0.16	E_music	0.3
k300_69	*elvi, presley, his, he, king, fan, memphi, graceland, rock, roll, parker, year, priscilla, record, death*	5	954	Topic_197	0.19	E_music	0.28

Table S25 – Topic description: Elvis Presley (correlation matrix)

	k100_78	k200_46	k300_69
k100_78	1		
k200_46	0.82	1	
k300_69	0.83	0.97	1

Table S26 – Topic description: 5G technology

**K_Topic**	**Top 15 words**	**N C**	**N M**	**Max Topic**	**r**	**Max LF**	**r**
k100_16	*5g, technolog, network, system, data, devic, comput, mobil, phone, use, digit, huawei, new, connect, communic*	393	1449	Topic_036	0.11	E_computer	0.51
k200_41	*5g, network, mobil, technolog, huawei, wireless, phone, connect, servic, compani, communic, speed, equip, infrastructur, telecom*	144	901	Topic_061	0.15	E_technology	0.34
k300_243	*5g, network, mobil, technolog, huawei, wireless, connect, servic, phone, compani, communic, telecom, speed, internet, secur*	128	851	Topic_252	0.18	E_technology	0.33

Table S27 – Topic description: 5G technology (correlation matrix)

	k100_16	k200_41	k300_243
k100_16	1		
k200_41	0.84	1	
k300_243	0.83	0.98	1

7.1 Topic specificity across different *k*s

Different ks offer different topic resolutions, where the higher the k (*i.e.*, the highest number of topics within the corpus) the higher the topic resolution (it follows that lower k offers a more general topic description). For example, within the topics around the MH370 flight disappearance, *i.e.*, topics k100_51, k200_142, and k300_272 (see Tables S12 and S13), it is possible to observe how topic specificity increases as a function of k. First, the keyword “mh370” moves from position 15 (in k100) to the 6th (k200) and 4th (k300) position indicating higher beta value for such term hence higher weight in the topic, which, from k100 to k300 describes more specifically the MH370 disappearance rather than flights in general. Second, the number of documents where this is the top topic (*i.e.*, higher gamma value across all topics within k dataset) decreases, indicating indeed higher specificity. Lastly, the correlations with the general lexical feature of *air_travel* decrease with the increase of k, suggesting that the topic is less general, focusing less on *air_travel* and more specifically on MH370. An increase in topic specificity as a function of k is visible in the Figure S4 below. For each of the three topics related to the disappearance of the flight MH370, we plotted the gamma values of all documents across a time span ranging from 1995 to 2020. We consider as more specific the topics that have less noise (*i.e.*, lower values) in gamma values in the period that precedes the event on the date 8th March 2014.

Figure S4 – The same topic over time on different ks

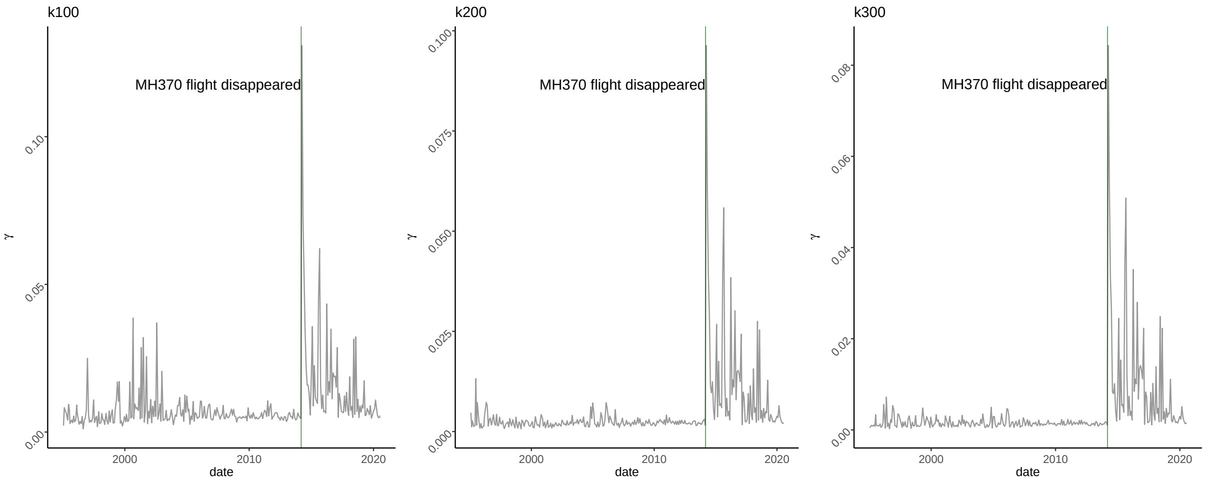

SM 8 Representativeness of conspiracy documents

In the Table S28 and Table S29 below, we show the top/bottom five conspiracy representative documents (first 1,000 characters) ordered by the cosine similarity value.

Table S28 – Top 5 highest representative conspiracy documents

doc_id	cs	text
C01b90	0.280	The reality of what's truly happening inside the United States is far, far more terrifying than just being screwed. Because quite literally, they are trying to KILL US! "Oh, come on now," I can hear you thinking. "Get real!" Well that's exactly what I'm will be doing in this article. I'm going to get very real. I hope that you'll read all the way to the bottom because you really need to know what's happening and why this isn't just a big government stupidity at work. What I share here is more than deadly serious and it is based upon a lot of research that many others have put together to clarify what's really happening in our country. Be sure to check out some of the videos I've included on this page to confirm what I'm telling you is true. This is hard for us to hear. It is difficult for rational and sane individuals to believe that anything on this scale could actually be happening around the world. That's why its so easy to dismiss this, without bothering to really
C0274e	0.280	Why are they trying to kill us? A deeper look into the mass production of America's toxic food My illustration on the left is really much too tame. I don't mean to imply that you'll just have to live with stupid government stunts, as if the screw they're turning into our collective chests might be only a painful inconvenience. The reality of what's truly happening inside the United States is far, far more terrifying than just being screwed. Because quite literally,they are trying to KILL US! "Oh, come on now," I can hear you thinking. "Get real!" Well that's exactly what I'm will be doing in this article. I'm going to get very real. I hope that you'll read all the way to the bottom because you really need to know what's happening and why this isn't just a big government stupidity at work. What I share here is more than deadly serious and it is based upon a lot of research that many others have put together to clarify what's really happening in our country. Be sure to check out so HT1
C05196	0.277	The great unraveling is gaining momentum by the day, and even now the majority of the masses are fast asleep at the wheel. Our skies look like something from an apocalyptic future, our trees are dying, our oceans are dying, our planet is dying, and still the majority remain unaware. Those in power are utilizing every option at their disposal to keep populations from waking until the last possible moment. Is there still time to change course and keep any part of the ship floating? That remains to be seen, but if there is yet a chance, it will require the concerted, focused, effective, prioritized, and completely dedicated efforts of all those that are already awake. The essay below was penned for geoengineeringwatch.org by a former USAF communications officer, Col. Randall Smith, Ph.D. Though I have no means of conclusively confirming the information presented by Randall, available date does generally confirm his estimations and conclusions. The essay does not address the implosion of t
C00113	0.276	Q: If Donald Trump, the Commander in Chief of the US Military, is Historically, Theologically and Morally Blind and Also Scientifically Illiterate, Should He be Making Life and Death Decisions for the Planet? "We have grasped the mystery of the atom and rejected the Sermon on the Mount. Ours is a world of nuclear giants and ethical infants. We know more about war than we do about peace – more about killing than we do about living." — WWII General Omar Bradley "If (Japan does) not now accept our terms they may expect a rain of ruin from the air, the likes of which has never been seen on this earth." – US President Harry S. Truman (August 6, 1945) "North Korea best not make any more threats to the United States... (If they do) they will be met with fire and fury... the likes of which this world has never seen before." – US President Donald J. Trump (August 8, 2017) On the eve of the anniversary of the United State's nuclear annihilation of the Christian community of Nagasaki on Aug
C036c7	0.276	Still Believe the New World Order Is Just A Conspiracy Theory? people say that they understand what the New World Order and a One World Government is all about, but that it doesn't necessarily have to be a bad thing. Maybe we need a New World Order, where we can live in one global state, in peace with no future wars? If there are no other countries to fight against, there will be no more wars either, right? And perhaps we will feel more united, as One Big Global Family if we erase the borders? Well, that doesn't sound bad, does it? If the intention behind the New World Order was the above, I would agree (although I don't think a centralized power can ever work. What if the Global CEO doesn't have our best interests in mind?). So, is the above utopia what the Globalists have in mind? Is this benevolent World Society what they so eagerly work towards? The best way to answer those questions is to quote from "The horse's mouth" so to speak. What do the Globalists have to say themse

Note. doc_id = LOCO's unique document ID; cs = cosine similarity.

Table S29 – Top 5 lowest representative conspiracy documents

doc_id	cs	text
C01782	0.002	More science reports have confirmed the engineered DNA of the new Corona virus. Earth and societal changes are accelerating at blinding speed on countless fronts, links in the chain of our current reality are already breaking down. In spite of unfolding biosphere collapse, the US stock market has hit yet another record high. How long can normalcy bias be maintained in first world populations? What happens when the gravity and immediacy of all that is unfolding can no longer be hidden from public consciousness? The latest installment of Global Alert News is below. As the horizon continues to darken, many are choosing to double down on denial and apathy. In contrast, ever increasing numbers are awakening and choosing a path of courage and action toward the greater good. We must never underestimate our collective power if we stand together. All are needed in the critical battle to wake populations to what is coming, we must make every day count. Share credible data from a credible sour
C01234	0.023	Rothschild inherited some very important patents when flight MH-370 disappeared When Malaysia Airlines Flight MH370 went missing, a US tech firm had 20 senior staff on the flight, many of whom were shareholders in the company and some were co-owners of some very important patents for military radar systems and microchips for autonomous driving cars. With the mysterious disappearance of the Boing 777, all of the remaining shares held by the missing travelers for Freescale Semiconductor Ltd were inherited by the only remaining shareholder, Lord Jacob Rothschild, who then became the sole owner of some very important patents. PayPal: Donate in USD PayPal: Donate in EUR PayPal: Donate in GBP
C01607	0.025	Here is the chemtrails patent: "A method is described for reducing atmospheric or world-wide warming resulting from the presence of heat-trapping gases in the environment, *i.e.*, from the greenhouse impact. This kind of gases are relatively transparent to sunshine, but absorb strongly the extended-wavelength infrared radiation released by the earth. The method incudes the phase of seeding the layer of heat-trapping gases in the environment with particles of materials characterized by wavelength-dependent emissivity. Such materials include Welsbach components and the oxides of metals which have higher emissivity (and as a result minimal reflectivities) in the visible and 8-twelve micron infrared wavelength areas."
C02dc1	0.030	As a supposed act of defiance, The Institute of Digital Archaeology, Harvard University, and UNESCO are erecting 43 foot tall 23 foot wide Arches of the Temple of Baal in New York's Times Square and London's Trafalgar Square. The date to unveil the arches falls directly on the celebration of the all important pagan holiday Beltane, and anniversary of the massacre of The Branch Davidians at WACO and The Oklahoma City Bombing, April 19. Related: Will A Gateway Be Opened When The Arch From The Temple Of Baal Is Reconstructed In Times Square? The Infowars Life Lung Cleanse Plus is back in stock at 50% off with double Patriot Points and free shipping!
C00b0a	0.031	WEBINAR: Dead Sea Scrolls show Sanhedrin may have executed Jesus as an ET Disclosure activist – Peter Kling & Alfred Lambremont Webre Dr. Frank Stranges lecture on the Dead Sea scrolls in which he reads from the many open descriptions of UFO, spaceships, other planets and Extraterrestrials, outer space battles, in the Dead Sea Scrolls, and the death threats that Dr. Frank Stranges received from the Israel-based Rabbinical guardians of the Scrolls for making these contents public! Is the Israel the faction behind the national security guardianship of the Dead Sea Scrolls in fear of being exposed by the ETs with the return of Jesus, who is an ET! IMHO – As Stranges illustrates from the guarded Dead Sea Scrolls. Here is the late, great Dr. Stranges bio:

Note. doc_id = LOCO's unique document ID; cs = cosine similarity.

SM 9 Popularity and spread metrics correlations

Besides single URLs shares for each webpage, we also obtained three other metrics for websites shares and compared them. This was done by: a) entering on SharedCount.com (SC) the website domain (*i.e.*, home page, *e.g.*, `www.infowars.com/`); b) computing the sum of all LOCO documents' URLs shares per website; c) computing an estimation of website total shares based on our observed shares in LOCO's documents (see main text for details, sections 3.8.4 and 3.8.6).

Below, in Table S30 (and in Figure S5) we show the correlation matrix (and scatter plots) for the main spread and popularity measures (all variables are log transformed). After having obtained the website shares from SC (believing it was a measure of overall website shares, see FB_shares_homepage in `website_metadata.json`), we soon realized that, although it correlates with both website rank (r = -.58) and monthly visits (r = .61), this measure does not reflect the total website shares. In fact, when we subtracted the computed sum of all URLs shares to this measure, we obtained a large number of negative values, meaning that the sum of the single URLs was shared more times than the overall website (*i.e.*, homepage). This led us to re-think this measure and in fact interpret it not as a measure of the whole website shares but as a measure of shares of the website's homepage. Although we do not see this measure as useful, we keep it in LOCO to allow users to replicate our analyses or use it for testing hypotheses we have not envisioned so far.

Table S30 – Correlation matrix of spread measures

Variable	1	2	3	4
1. Global rank				
2. Total visits	-.99			
	[-.99, -.99]			
3. FB shares homepage	-.58	.61		
	[-.69, -.45]	[.48, .71]		
4. LOCO sum shares	-.36	.37	.68	
	[-.51, -.20]	[.20, .51]	[.58, .76]	
5. FB shares projected	-.81	.81	.77	.85
	[-.86, -.73]	[.74, .86]	[.70, .83]	[.80, .89]

Note. Global rank: websites' global rank (from SimilarWeb); Total visits: websites' monthly visits; FB shares homepage: number of times the websites' homepage is shared on Facebook; LOCO sum shares: the aggregated number of individual LOCO's webpages shares on Facebook; FB shares projected: the computed number of overall Facebook shares given observed data in LOCO. Values in square brackets indicate the 95% confidence interval for each correlation. All correlations are significant at $p < .01$.

Figure S5 – Scatter plots of spread measures

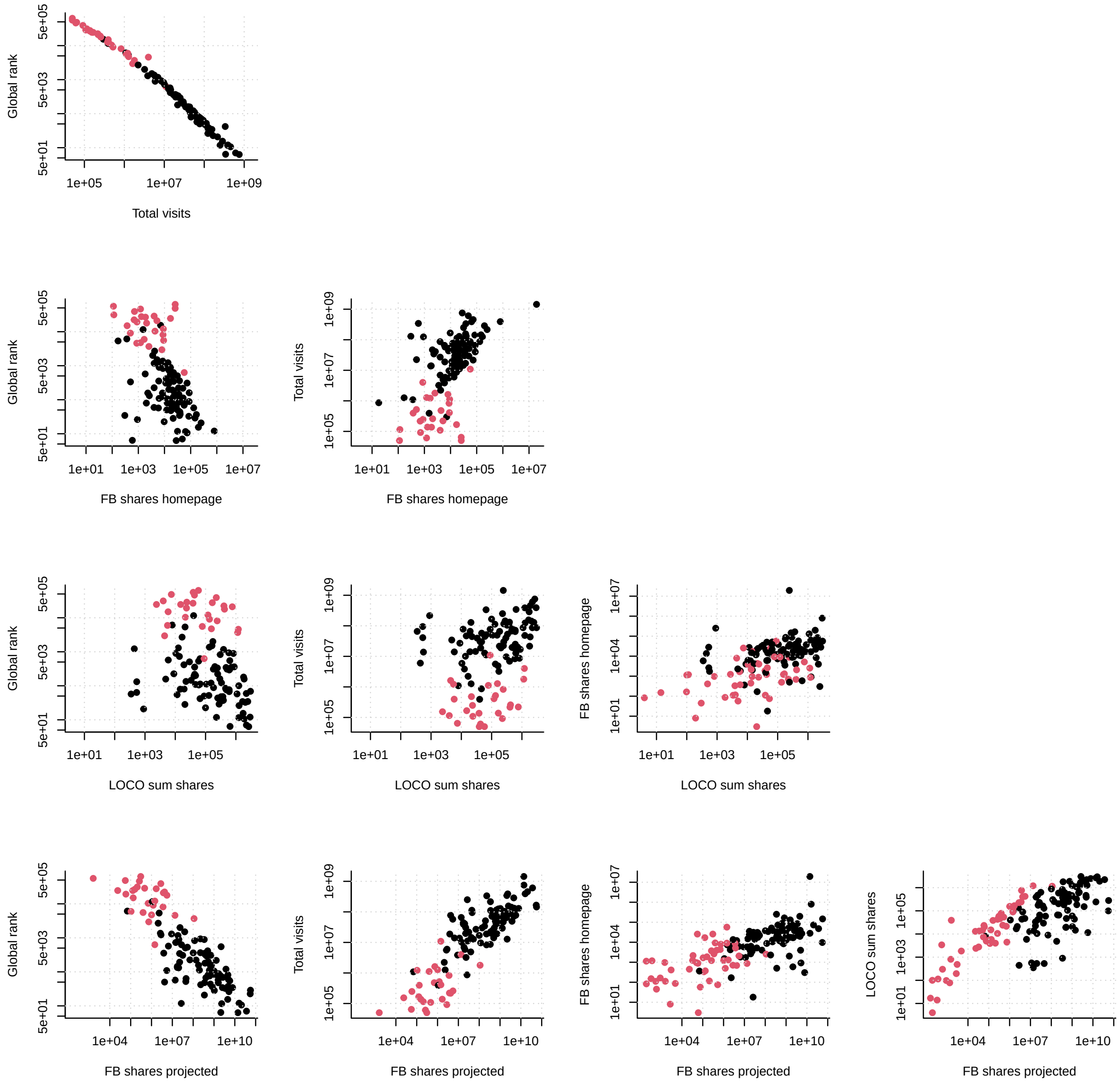

Note. Red and black dots represent conspiracy and mainstream websites respectively. Global rank: websites' global rank (from SimilarWeb); Total visits: websites' monthly visits; FB shares homepage: number of times the websites' homepage is shared on Facebook; LOCO sum shares: the aggregated number of individual LOCO's webpages shares on Facebook; FB shares projected: the computed number of overall Facebook shares given observed data in LOCO.

SM 10 Lexical features of mentioning conspiracy

In section 4.2.1 of the main text, we have shown how LOCO's lexical features overlap with those obtained in Klein et al. (2019) from Reddit. Here, with the same protocol and on the same set of Empath categories, we explored the effect of cleaning the mainstream subcorpus from mentions to conspiracy (see section 3.8.1 of the main text). As shown in Figure S6, overall, the absolute effect size is larger when mainstream documents mentioning conspiracy are removed (green error bars) compared to the mainstream subcorpus that includes all documents (red error bars).

We then tested whether changes in effect size from the corpus with all documents (raw data) to the corpus without documents mentioning conspiracy (cleaned data) were significant and so we ran a paired samples t-test on the absolute d values. The t-test showed a significant increase in effect size from raw to clean data, $t_{30} = 5.08$, $p < .001$, see Figure S7 below.

We then took the top four categories that yielded the larger changes in effect sizes and tested whether there was a correlation between the number mentioning conspiracy and those four lexical features. Results with log-transformed variables (plots in Figure S8) showed a positive relationship: crime: $r = .31$; terrorism: $r = .33$; deception: $r = .21$; and stealing: $r = .13$ (Non log-transformed variables: deception: $r = .14$; terrorism: $r = .25$; crime: $r = .20$; stealing: $r = .06$).

Figure S6 – Effect of mentioning conspiracy on lexical features

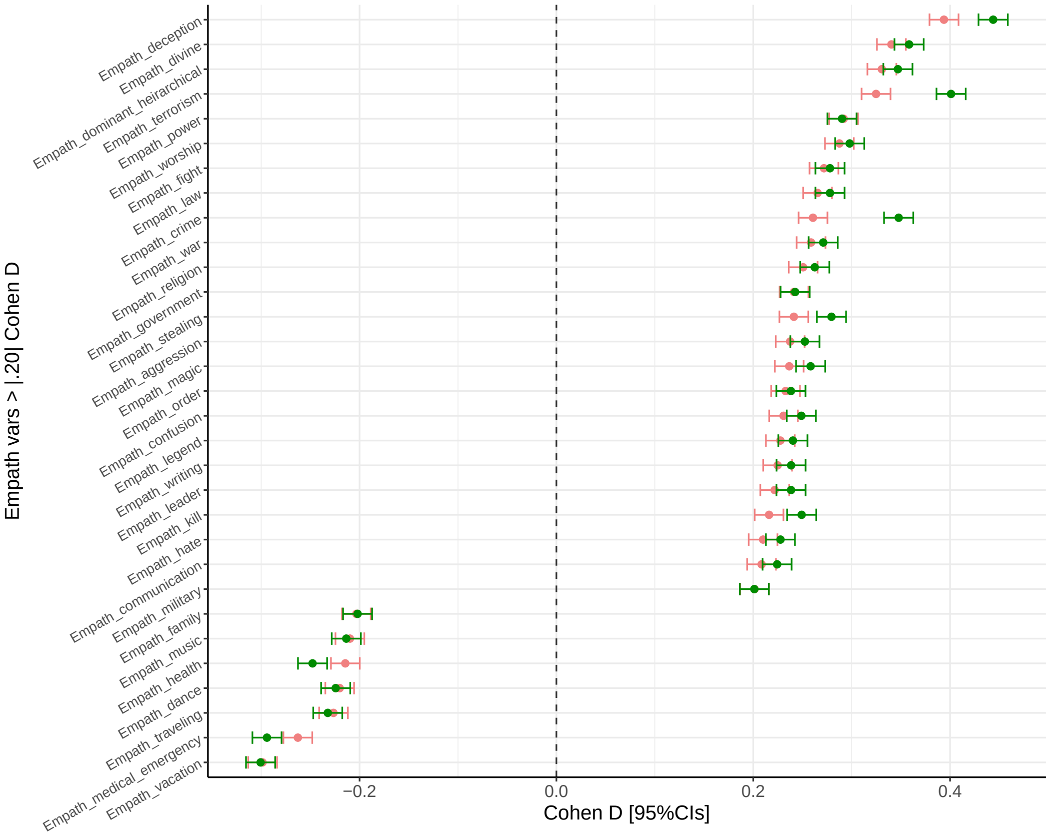

Note. Differences, in Cohen's d, between conspiracy and all mainstream documents (red error bars, raw data) and between conspiracy and mainstream documents without documents mentioning conspiracy (green error bars, cleaned data). Positive values indicate that the effect is higher in conspiracy.

Figure S7 – Paired t-test on effect size changes from raw to cleaned subcorpora

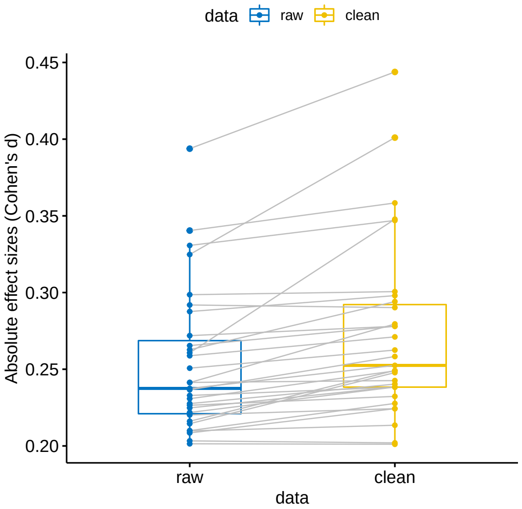

Figure S8 – Scatter plots of number of mentions of conspiracy and lexical features

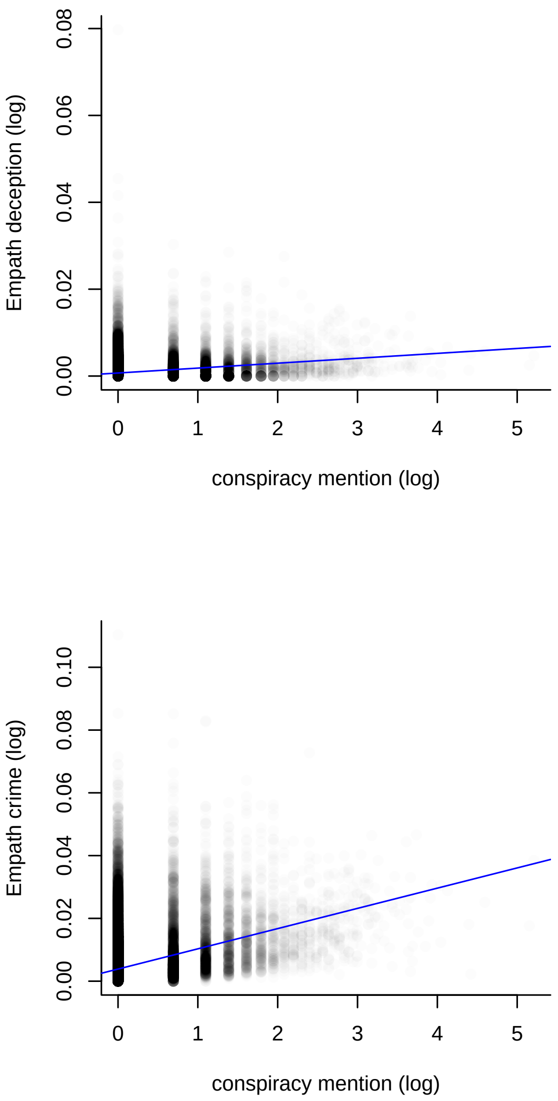

Note. All variables have been log-transformed prior to analyses

SM 11 Properties of representative conspiracy documents

In section 4.2.3 of the main text, we have plotted the Standardized beta estimates of multilevel models in which we specified as random intercept the topics obtained setting k at 100. Here, we replicate the same analyses changing instead the random intercept with topic obtained at $k = 200$. Results are not observably different (compare with Figure 4 in the main text). See Figure S9.

Figure S9 – Differences in lexical features between high and low representative conspiracy documents

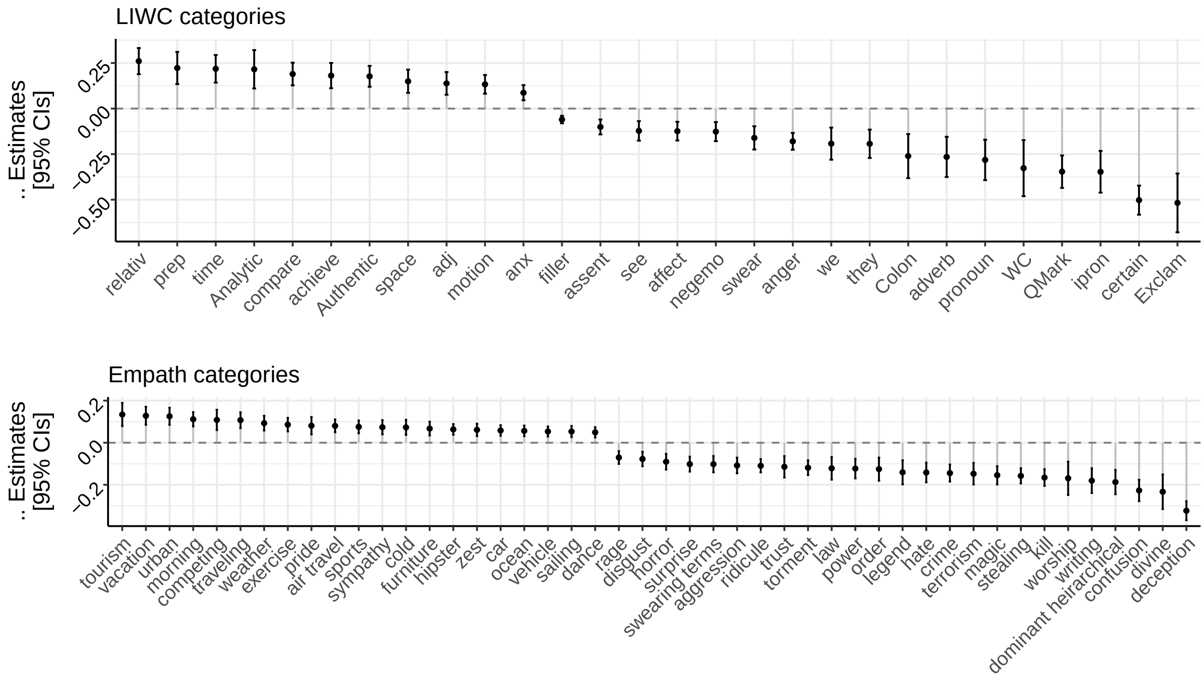

References

Benoit, K., Muhr, D., and Watanabe, K. (2020). stopwords: Multilingual Stopword Lists.

Benoit, K., Watanabe, K., Wang, H., Nulty, P., Obeng, A., Müller, S., and Matsuo, A. (2018). quanteda: An R package for the quantitative analysis of textual data. *Journal of Open Source Software*, 3(30):774.

Douglas, K. M., Uscinski, J. E., Sutton, R. M., Cichocka, A., Nefes, T., Ang, C. S., and Deravi, F. (2019). Understanding Conspiracy Theories. *Political Psychology*, 40(S1):3–35.

Fry, E. (2000). *1000 instant words: the most common words for teaching reading, writing and spelling*. Teacher Created Resources.

Klein, C., Clutton, P., and Dunn, A. G. (2019). Pathways to conspiracy: The social and linguistic precursors of involvement in Reddit's conspiracy theory forum. *PLOS ONE*, 14(11):e0225098.

Nguyen, D., Liakata, M., DeDeo, S., Eisenstein, J., Mimno, D., Tromble, R., and Winters, J. (2020). How We Do Things With Words: Analyzing Text as Social and Cultural Data. *Frontiers in Artificial Intelligence*, 3.

Oswald, S. (2016). Conspiracy and bias: argumentative features and persuasiveness of conspiracy theories. *OSSA Conference Archive*, 168:1–16.

Rinker, T. W. (2013). qdapDictionaries: Dictionaries to Accompany the qdap Package.
